# Chimeric Nanozyme Bacterial Outer Membrane Vesicles Reprograming Tumor Microenvironment for Safe and Efficient Anticancer Therapy

**DOI:** 10.1002/advs.202417712

**Published:** 2025-04-25

**Authors:** Fan Zhang, Qianqian Li, Haibing Dai, Weiqun Li, Xiang Chen, Huibin Wu, Shanming Lu, Ran Luo, Feng Li, Guihong Lu, Jianbo Yu, Lin Mei

**Affiliations:** ^1^ Center for Child Care and Mental Health (CCCMH) Shenzhen Children's Hospital Shenzhen Guangdong 518038 China; ^2^ Longgang Central Hospital Shenzhen Guangdong 518100 China; ^3^ State Key Laboratory of Advanced Medical Materials and Devices Tianjin Key Laboratory of Biomedical Materials Key Laboratory of Biomaterials and Nanotechnology for Cancer Immunotherapy Institute of Biomedical Engineering Chinese Academy of Medical Sciences and Peking Union Medical College Tianjin 300192 China; ^4^ Institute of Pharmaceutics Shenzhen Campus of SunYat‐sen University Shenzhen Guangdong 518107 China; ^5^ Shenzhen Bay Laboratory Shenzhen Guangdong 518132 China; ^6^ Key Laboratory of Biopharmaceutical Preparation and Delivery Chinese Academy of Sciences Beijing 100190 P. R. China

**Keywords:** ferroptosis, immunomodulation, nanozyme, outer membrane vesicles, tumor

## Abstract

This study presents an innovative approach utilizing a biocompatible shell to shield bacterial outer membrane vesicles (OMVs) and incorporate Fe ions and ultrasmall Au nanoparticles to develop a combined tumor therapeutic strategy. These chimeric nanozyme shells effectively reduce the toxicity of OMVs during circulation and promote their accumulation in tumor tissues. In the tumor microenvironment, Au nanoparticles act as nanozymes, catalyzing glucose consumption and elevating H₂O₂ levels. The increased H₂O₂ subsequently reacts with the released Fe ions to induce immunogenic tumor cell death through iron‐mediated chemodynamic mechanisms. Simultaneously, the release of tumor‐associated antigens and OMVs synergistically stimulates the immune response. This cascade of nanozyme‐catalyzed reactions, chemodynamic effects, and immune activation results in efficient tumor inhibition.

## Graphical abstract

This study reports an innovative strategy to improve the safety of OMVs by shielding them with a sophisticated shell composed of DaFe shell and Au nanoparticles, which not only reduces the toxicity of OMVs during circulation but also enables rapid release of these therapeutically active components in tumor for combined therapy involving glucose consumption, chemodynamic therapy, and immunotherapy.

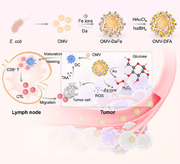

## 1 Introduction

Bacteria‐based formulations exhibit unique advantages over conventional biomaterials in tumor therapy.^[^ ^1^, ^2^, ^3^ ^]^ For example, bacterially derived minicells and outer membrane vesicles (OMVs) possess rigid membranes that enhance stability and minimize leakage during systemic circulation, making them highly promising nanocarriers.^[^ ^4^, ^5^ ^]^ Notably, OMVs naturally contain various immunostimulatory components, including lipopolysaccharide (LPS), outer membrane proteins, lipoproteins, and nucleic acids derived from their parent cells. These components potentiate specific antitumor responses by promoting adaptive immunity.^[^ ^6^, ^7^, ^8^ ^]^ Additionally, OMVs can be efficiently produced in large quantities through well‐established fermentation and purification processes.^[^ ^9^, ^10^ ^]^ However, the presence of LPS and other bacterial cell wall components in OMVs can trigger acute toxicity, posing significant challenges for their application in cancer immunotherapy.^[^ ^11^, ^12^ ^]^ This underscores the urgent need for engineered OMV‐based formulations that enhance tumor accumulation, cytotoxicity, and immune activation while mitigating systemic toxicity.

Structural modifications of OMVs have shown promise in reducing severe systemic inflammation caused by unmodified vesicles. The shielding of toxic agents by encapsulation strategies is particularly effective.^[^ ^13^, ^14^, ^15^ ^]^ Among these approaches, calcium phosphate (CaP) encapsulation of OMVs with a biocompatible, pH‐sensitive nanoshell has demonstrated significant antitumor efficacy with minimal side effects.^[^ ^16^ ^]^ Despite these advancements, the efficacy of modified OMVs in shielding against toxic agents remains suboptimal, particularly in the treatment of solid tumors. Given the robust immune activation potential of OMVs, their combination with tumor immunogenic cell death (ICD) mechanisms offers a compelling strategy to enhance tumor inhibition.^[^ ^17^, ^18^ ^]^ Within the acidic tumor microenvironment (TME), Fe^2^⁺ ions react with H₂O₂ to generate cytotoxic hydroxyl radicals (•OH) through the Fenton reaction, thereby inducing tumor cell death and supporting chemodynamic therapy (CDT).^[^ ^19^, ^20^ ^]^ However, the limited availability of H₂O₂ in tumors often restricts the effectiveness of CDT, necessitating strategies to enrich H₂O₂ within the TME.

Encouragingly, enzymes capable of catalyzing glucose to gluconic acid and H₂O₂ offer an in situ solution for increasing H₂O₂ levels within tumors.^[^ ^21^, ^22^, ^23^ ^]^ However, natural enzymes face challenges such as low stability, difficult scalability, and storage limitations, which hinder their broader application in cancer therapy.^[^ ^24^, ^25^ ^]^ As a result, research efforts have focused on developing nanozymes—nanotechnology‐enabled analogs of natural enzymes. Nanozymes, including those with peroxidase (POD)‐like, glucose oxidase (GOx)‐like, and superoxide dismutase (SOD)‐like activities, offer advantages such as enhanced stability and cost‐effectiveness.^[^ ^26^, ^27^, ^28^ ^]^ Among various nanozymes, gold nanoparticles (AuNPs) have garnered particular interest because of their high efficiency and multifunctional enzyme‐like activities, including GOx‐like catalytic properties, which enable the oxidation of glucose to gluconic acid while consuming oxygen.^[^ ^29^, ^30^, ^31^, ^32^ ^]^

**Scheme 1:**
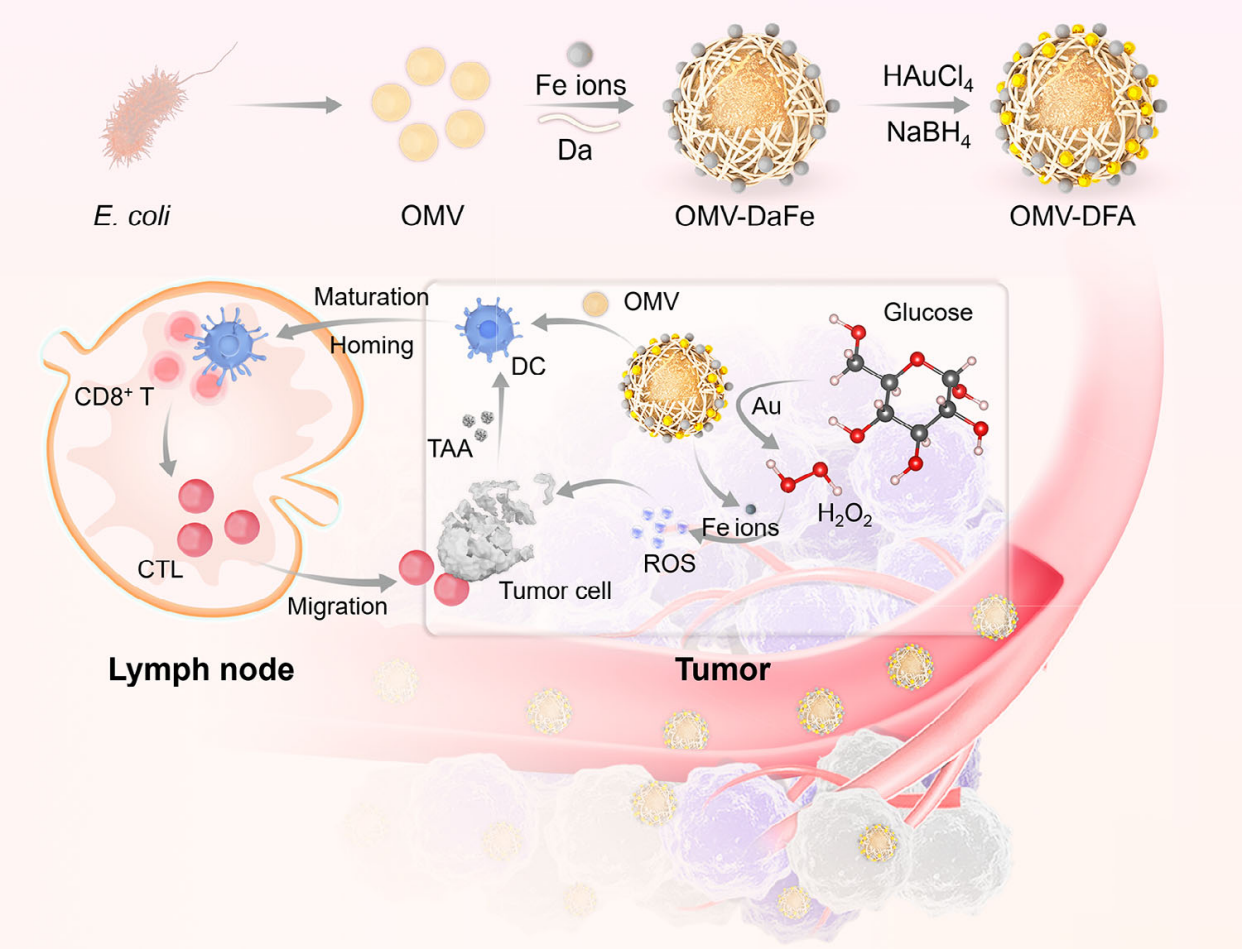
Schematic illustration of preparation procedure of OMV‐DFA and its cascade effect for cancer therapy. The OMV‐DFA would accumulate in tumor microenvironment after intravenous injection, which would mediate the H₂O₂ production through Au nano enzyme, generate ferroptosis with Fe ion, and induce immune activation with OMVs. The synergistically promotes tumor cell elimination, activates immune responses, reduces immune suppression, and maximizes treatment efficacy.

Herein, we designed and synthesized a novel nanoplatform by encapsulating bacterial OMVs with SH‐PEG‐Dopamine (Da) and Fe ions, followed by the in situ growth of ultrasmall gold nanoparticles (≈3 nm) on the surface, forming Da‐Fe‐Au‐armed OMVs (OMV‐DFA). This construct, enriched within the tumor microenvironment, functions as a multifunctional tumor immunotherapy system with a ternary cascade effect for tumor eradication (**Scheme** **1**). In this system, the coordination‐mediated interfacial interaction between ferric ions (Fe^III^) and phenol in Da facilitated the assembly of DaFe metal‐phenolic “network” on the OMV surface. Then, ultrasmall AuNPs were grown based on the strong interaction between gold ions and sulfhydryl (SH) groups in PEG molecules. This encapsulation reduces the systemic toxicity of OMVs. The subsequent growth of ultrasmall AuNPs on the PEG‐DaFe network facilitated a ternary cascade effect against tumors. Upon accumulation in the tumor, AuNPs catalyze the oxidation of glucose to gluconic acid, inducing starvation therapy (ST). Simultaneously, the H₂O₂ generated during Au‐mediated glucose oxidation and the acidic TME cooperatively promotes the depolymerization of the DaFe metal‐phenolic network, releasing Fe ions that react with H₂O₂ to produce cytotoxic •OH radicals for CDT. Together, ST and CDT induce ICD, releasing tumor‐associated antigens (TAA). These antigens, along with the immunogenic properties of OMVs, activated robust immune responses, alleviated immune suppression within the TME, and enhanced tumor destruction. This ternary cascade mechanism, integrating AuNP‐mediated ST, Fe‐ion‐mediated CDT, and OMV‐induced immune activation, synergistically inhibited tumor progression, metastasis, and recurrence.

## 2 Results and Discussion

### 2.1 Preparation and Characterization of OMV‐DFA

**Figure 1:**
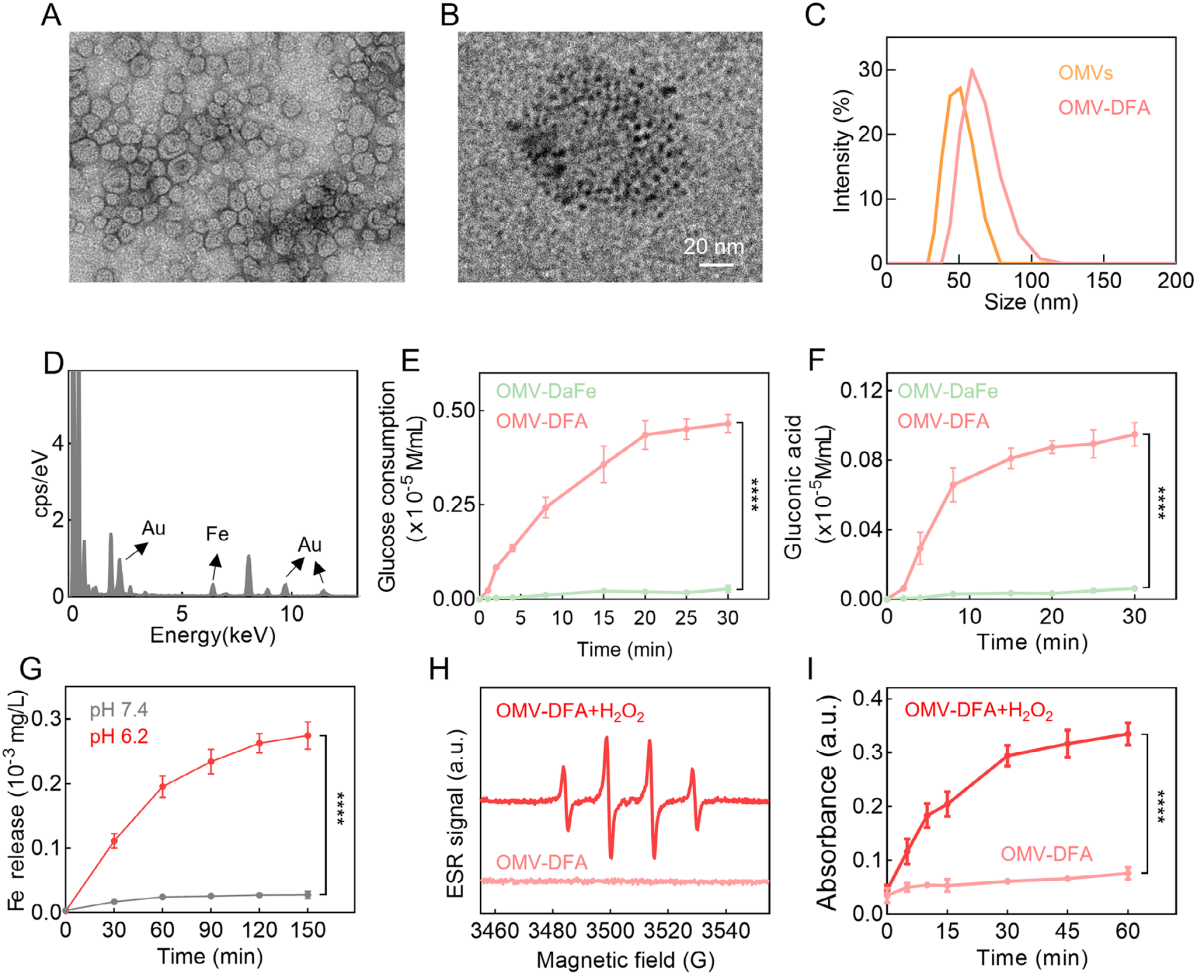
Construction and characterization of OMV‐DFA. (A,B) Representative TEM images of OMVs A) and OMV‐DFA B), showing spherical morphology and the presence of ultrasmall nano‐Au particles on OMV‐DFA. C) Hydrodynamic size distributions of OMVs and OMV‐DFA measured by dynamic light scattering (DLS). D) Energy‐dispersive X‐ray (EDX) spectrometry confirming the presence of Au and Fe elements on the OMV‐DFA. (E,F) Glucose consumption E) and gluconic acid production F) in glucose solutions incubated with OMV‐DaFe or OMV‐DFA over 30 min. G) Fe ion release profiles from OMV‐DFA solutions under physiological (pH 7.4) and weak acidic (pH 6.5) conditions. H) Electron spin resonance (ESR) spectra demonstrating •OH generation of OMV‐DFA in the presence of H_2_O_2_. I) Kinetics of •OH production of OMV‐DFA in the presence of H_2_O_2_. Data in E,F,G, and I are presented as the mean ± SD (*n* = 3). Statistical significance was assessed using a two‐tailed unpaired Student's *t*‐test. ^****^ *p* < 0.0001.

OMVs were isolated from *Escherichia coli* (BL21) following previously described protocols.^[^ ^33^ ^]^ The examination by transmission electron microscopy (TEM) revealed that the obtained OMVs were typical circular vesicle structure with a size of ≈50 nm (**Figure** **1**A). To minimize the potential side effects during circulation after intravenous injection, these OMVs were further encapsulated in a pH‐sensitive shell composed of Da and Fe ions to form OMV‐DaFe nanoparticles. Subsequent UV–vis–NIR optical absorption spectroscopy confirmed the successful encapsulation (Figure S1A, Supporting Information). To functionalize OMV‐DaFe with ultrasmall nano‐Au particles, Au ions were reduced by NaBH_4_ on the surface. Both TEM imaging and elemental mapping demonstrated the successful growth of ultrasmall nano‐Au particles (≈3 nm in diameter) on OMV‐DaFe (Figure 1B,D), thus obtaining OMV‐DFA. The construction of OMV‐DFA was further verified by its increased hydrodynamic diameter (Figure 1C). Further, sodium dodecyl sulfate‐polyacrylamide gel electrophoresis (SDS‐PAGE) and proteomics analysis showed comparable proteins in OMVs and OMV‐DFA, indicating that the DaFe encapsulating and nano‐Au growth had no significant influence on membrane proteins of OMVs (Figure S1B,C and Table S1, Supporting Information).

Given the goal of introducing ultrasmall‐nano Au particles for catalyzing glucose consumption, we incubated OMV‐DaFe or OMV‐DFA in a glucose solution and measured both glucose consumption and gluconic acid generation. As shown in Figure 1E,F, OMV‐DFA significantly outperformed OMV‐DaFe in terms of both glucose consumption and gluconic acid generation over time, demonstrating the high‐performance catalytic effect of ultrasmall nano‐Au particles on OMV‐DFA. In the acidic tumor microenvironment (TME), the pH‐sensitive shell of OMV‐DFA was supposed to disintegrate and release Fe ions for CDT. To confirm this, we first detected the Fe ions released from OMV‐DFA under different pH conditions and then evaluated hydroxyl radical (•OH) production during OMV‐DFA incubation with H_2_O_2_. Compared to pH 7.4, at which OMV‐DFA remained stable, abundant Fe ions were released under a TME‐mimic acidic condition (Figure 1G). Further electron spin resonance (ESR) and methylene blue bleaching assays indicated that the Fe ions released from OMV‐DFA could effectively react with H_2_O_2_ to produce •OH (Figure 1H,I), suggesting that OMV‐DFA has CDT potential in the TME. Additionally, OMV‐DFA demonstrated good storage stability, with no significant changes in particle size and Fe‐Au contents after one week of storage in phosphate‐buffered saline (PBS) (Figure S1D,E, Supporting Information).

### 2.2 Evaluation of Synergistic Antitumor Efficacy of OMV‐DFA In Vitro

**Figure 2:**
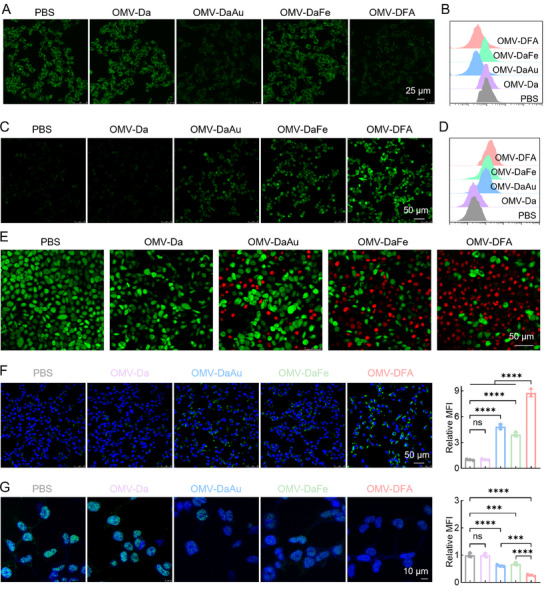
In vitro evaluation of the synergistic therapeutic effects of OMV‐DFA in 4T1 cells. A) Intracellular glucose content in 4T1 cells incubated with 2‐NBDG following treatment with PBS, OMV‐Da, OMV‐DaAu, OMV‐DaFe, or OMV‐DFA. B) Flow cytometry histograms illustrating glucose fluorescence signals in 4T1 cells after the indicated treatment. (C,D) Confocal laser scanning microscopy (CLSM) images C) and flow cytometry histograms D) of ROS level in 4T1 cells after various treatments. E) Live/Dead staining analysis of 4T1 cells subjected to the indicated treatments. Live cells were labeled with calcein‐AM (green), while dead cells were stained with ethidium homodimer‐1 (red). F) CLSM images and quantitative analysis of CRT expression in 4T1 cells after treatment with the indicated formulations. Green fluorescence represents CRT, and blue fluorescence corresponds to Hoechst 33342‐labeled nuclei. G) CLSM images and quantitative analysis of HMGB1 in the nuclei of 4T1 cells receiving the indicated treatment. Green fluorescence represents HMGB1, and blue fluorescence corresponds to Hoechst 33342‐labeled nuclei. Data in F and G are presented as the mean ± SD (*n* = 3). Statistical significance was assessed using one‐way ANOVA. ^***^ *p* < 0.001, ^****^ *p* < 0.0001, ns: not significant.

Having successfully obtained OMV‐DFA, we next evaluated the cascade of therapeutic effects using a murine breast cancer cell line (4T1). First, we utilized a glucose uptake and transport probe (2‐deoxy‐2‐[(7‐nitro‐2,1,3‐benzoxadiazol‐4‐yl) amino]‐D‐glucose, 2‐NBDG) to assess glucose consumption in 4T1 cells receiving the indicated treatment. Compared with the PBS group, the OMV‐Da and OMV‐DaFe groups presented an unchanged intracellular fluorescence signal of glucose, whereas OMV‐DaAu and OMV‐DFA treatments significantly reduced the intracellular fluorescence signal of glucose, suggesting that the superficial ultrasmall nano‐Au particles could effectively consume glucose contents (**Figure** **2A,B**; Figure S2A, Supporting Information). In terms of CDT effects, we detected ROS production in 4T1 cells using 2′,7′‐dichlorofluorescin diacetate (DCFH‐DA) as a probe. As revealed in the fluorescence images, the ROS signals in cells after incubation with OMV‐Da did not change compared to those in the PBS group (Figure 2C,D; Figure S2B, Supporting Information). OMV‐DaAu and OMV‐DaFe moderately increased ROS contents while OMV‐DFA abundantly generated ROS in cells, which was primarily due to the synergism of H_2_O_2_ generation from Au‐catalyzed glucose consumption and released Fe ion‐mediated chemodynamic effect. Such superior ROS generation capability of OMV‐DFA was further verified by flow cytometry analysis using hydroxyphenyl fluorescein (HPF) as specific •OH probe (Figure S3, Supporting Information).

The satisfactory glucose consumption and CDT effects of OMV‐DFA prompted us to investigate its therapeutic effect on cancer cells. The growth of cancer cells was only slightly inhibited by OMV‐DaAu (glucose consumption) or OMV‐DaFe (CDT), with over 70% of the cancer cells remaining alive after treatment (Figure 2E; Figure S4, Supporting Information). In contrast, the growth of cancer cells was statistically inhibited, and cell viability remained below 30%, indicating a superior therapeutic effect of the synergism of glucose consumption and CDT. Importantly, compared to other treatment groups, OMV‐DFA induced more calreticulin (CRT) expression and higher mobility group box 1 (HMGB1) release in 4T1 cells (Figure 2F,G), which effectively induced DC maturation and M1‐like macrophage polarization (Figure S5, Supporting Information), thus indicating immunogenic cell death (ICD) of cancer cells and highlighting the potential of combining immunotherapy in vivo.

### 2.3 Tumor Accumulation Performance of OMV‐DFA

**Figure 3:**
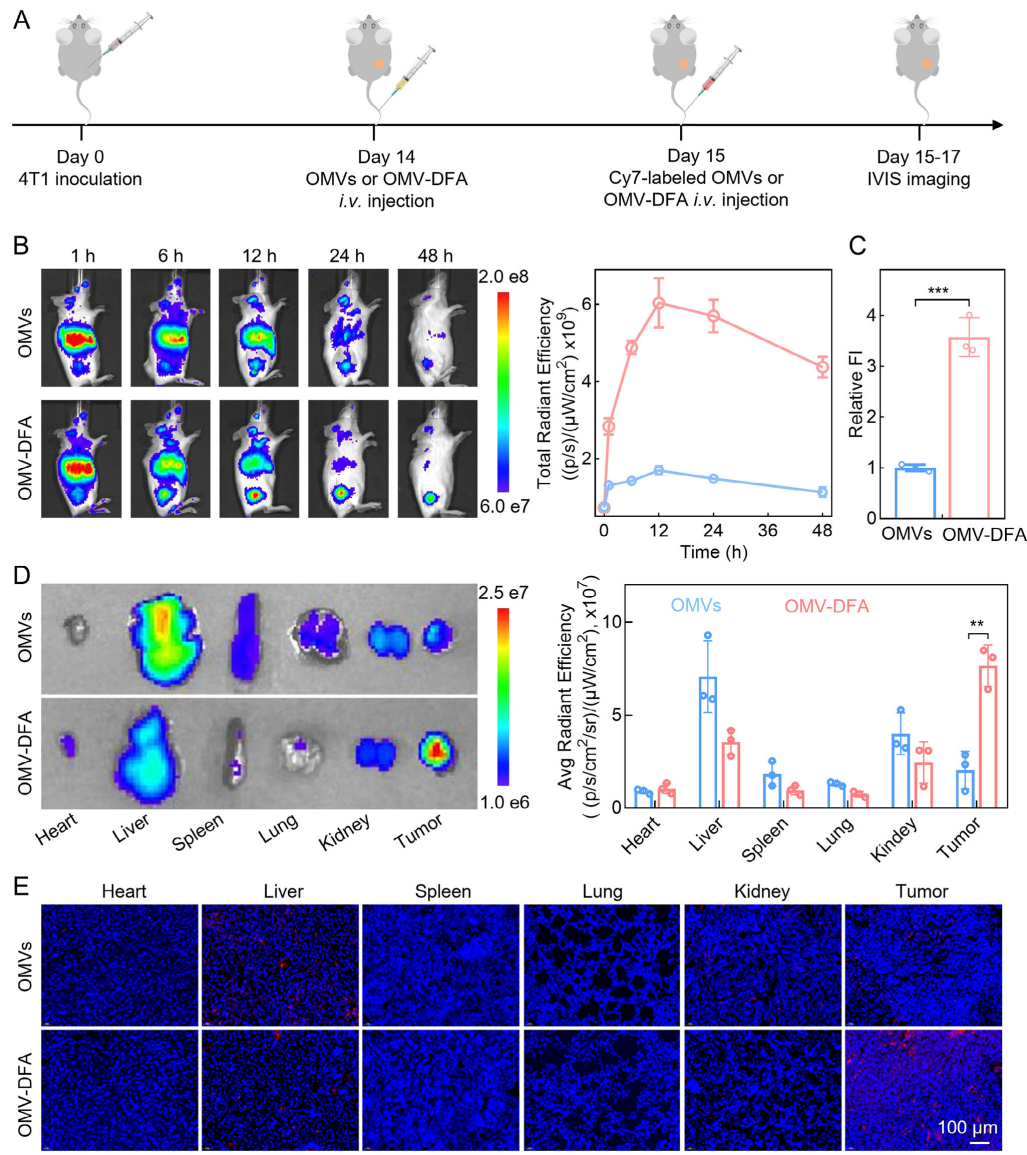
OMV‐DFA enables efficient tumor accumulation. A) Schematic illustration of the experimental design for evaluating the tumor accumulation of OMV‐DFA in 4T1 tumor‐bearing Balb/c mice. B) In vivo bioluminescence imaging and corresponding total radiant efficiency measurements of 4T1 tumors at various time points following intravenous injection of OMVs or OMV‐DFA. C) Relative tumor accumulation efficiencies were calculated from tumor fluorescence intensity (FI) at 12 h after intravenous injection of Cy7‐labeled OMVs or OMV‐DFA. D) Ex vivo fluorescence imaging of tumors and major organs collected from mice injected with Cy7‐labeled OMVs or OMV‐DFA, accompanied by quantitative analysis of fluorescence signals. E) Fluorescence imaging of tumor and organ sections from tumor‐bearing mice treated with OMVs or OMV‐DFA. Tumor and organ sections were labeled with DAPI for nuclei (blue) and Cy3 for OMVs or OMV‐DFA (red). Data in B, C, and D are presented as the mean ± SD (*n* = 3). Statistical significance was assessed using two‐tailed unpaired Student's *t*‐test. ^**^ *p* < 0.01, ^***^ *p* < 0.001.

Owing to their nano‐scale size, OMVs are theoretically capable of passively accumulating at tumor sites via enhanced permeability and retention (EPR) effects. A “shielding shell” has been reported to prevent the antibody‐dependent clearance of OMVs, thereby increasing their half‐life, and thereby enhancing tumor accumulation.^[^ ^33^ ^]^ To evaluate the shielding effect of our pH‐sensitive shell, we monitored the tumor accumulation of OMV‐DFA in 4T1 tumor‐bearing mice. When the tumor volume reached 150 mm^3^, we intravenously injected OMVs or OMV‐DFA on day 14 and Cyanine7 (Cy7)‐labeled OMVs or OMV‐DFA on day 15. Tumor targeting and in vivo distribution of both Cy7‐labeled formulations were determined using a IVIS imaging system at different time‐points (**Figure** **3**A). Although both OMVs and OMV‐DFA were detected at the tumor sites after administration, OMV‐DFA exhibited a 3.6‐fold higher tumor accumulation than did OMVs at 12 h post‐injection (Figure 3B,C; Figure S6A, Supporting Information). To further observe the distribution of OMVs and OMV‐DFA in vivo, mice in the parallel groups were sacrificed at 12 h after the second injection, and their tumors and main organs were collected for further evaluation by fluorescence imaging and histologic section analysis. Both sets of data revealed increased tumor accumulation and reduced liver uptake of OMV‐DFA compared to OMVs (Figure 3D,E; Figure S6B, Supporting Information), suggesting that encapsulating OMVs within DFA shells effectively enhanced passive tumor accumulation.

### 2.4 Therapeutic Effects of OMV‐DFA In Vivo

**Figure 4:**
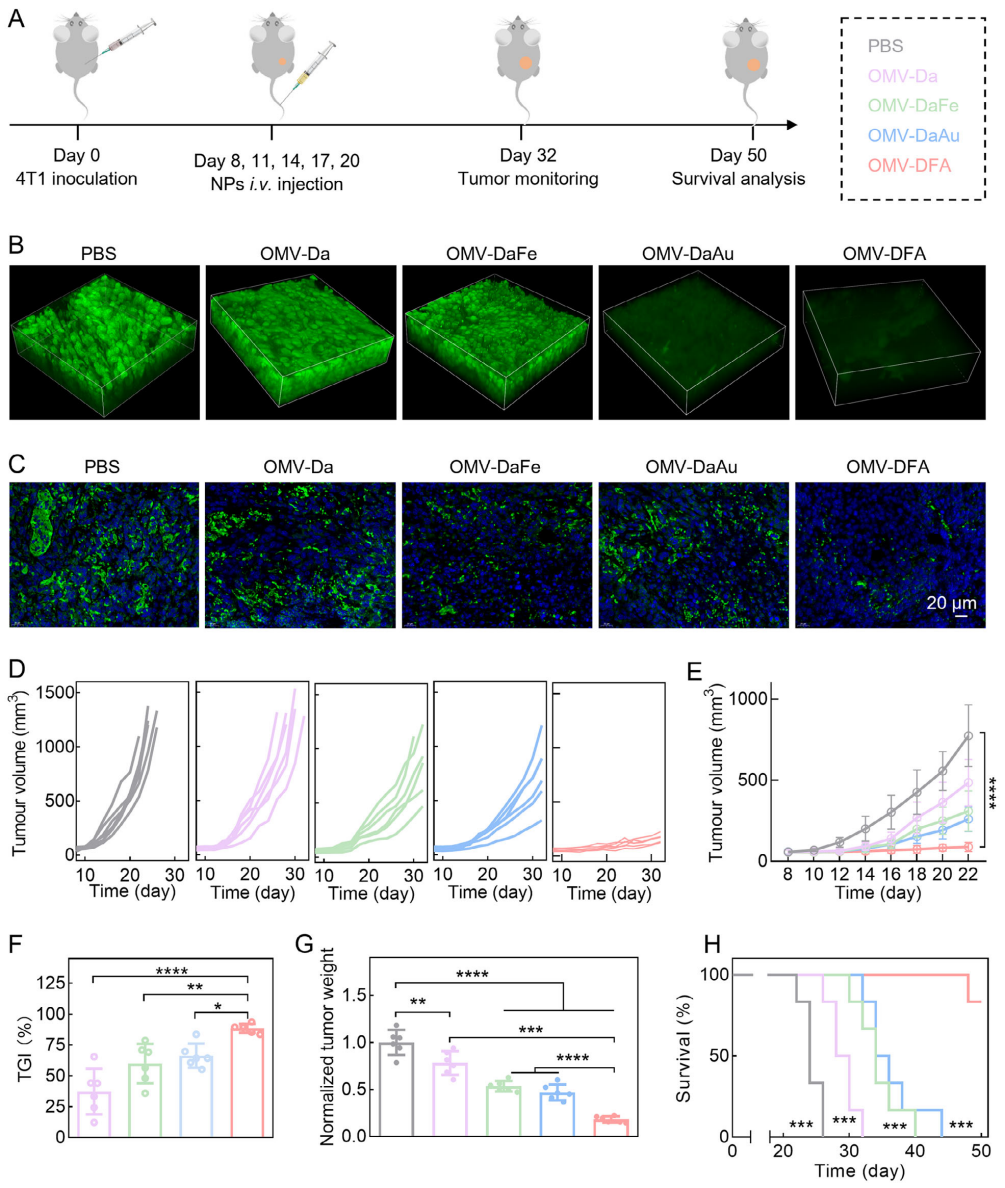
Evaluation of the cascade therapeutic effects of OMV‐DFA in vivo. A) Schematic representation of the therapeutic schedule for a 4T1 tumor model in Balb/c mice. Mice were subcutaneously inoculated with 4T1 cells and randomly assigned to five treatment groups: PBS, OMV‐Da, OMV‐DaFe, OMV‐DaAu, or OMV‐DFA. Treatments were administered via intravenous injections on days 8, 11, 14, 17, and 20. Mice were euthanized when primary tumor volumes exceeded 1000 mm^3^, and organs were harvested for analysis. B) Two‐photon fluorescence imaging of glucose uptake (2‐NBDG) in tumor tissues following various treatments (scale bar: 50 µm). C) Immunofluorescence imaging of GPX4 expression in tumor sections from 4T1‐bearing mice after treatment, highlighting differential therapeutic effects of the tested formulations. D) Individual tumor growth curves for each treatment group (*n* = 6). E) Tumor volumes were measured over time in the different treatment groups. (F,G) TGI rates F) and tumor weights (E) in each treatment group on day 22. (H) Kaplan‐Meier survival curves for 4T1 tumor‐bearing mice in each treatment group (*n* = 6). Data in E‐G are presented as the mean ± SD (*n* = 6). Statistical significance was assessed using one‐way ANOVA (E‐G) and Log‐rank tests (H). ^*^ *p* < 0.05, ^**^ *p* < 0.01, ^***^ *p* < 0.001, ^****^ *p* < 0.0001.

Building on the potent therapeutic effects (glucose consumption and CDT) in vitro and strong tumor accumulation ability in vivo, we subsequently studied the therapeutic performance of OMV‐DFA in vivo using a 4T1 xenograft tumor model (**Figure** **4**A). First, we evaluated the glucose consumption in tumors following intra‐tumoral administration of 2‐NBDG (Figure 4B; Figure S7, Supporting Information). We found that OMV‐DaAu and OMV‐DFA significantly reduced glucose levels compared to the other groups, indicating the superior glucose consumption‐ability of the superficial ultrasmall nano‐Au particles in the tumor. Then we evaluated the chemodynamic effects by detecting the expression of GPX4 (which resists ROS in cells) in the tumor (Figure 4C; Figure S8, Supporting Information). In line with our expectations, less GPX4 was detected in the tumors of mice receiving OMV‐DaFe treatment than in the PBS, OMV‐Da, and OMV‐DaAu groups, with yet further significant decrease of GPX4 expression in the OMV‐DFA group (over 2.9‐fold lower than that in the OMV‐DaFe group).

The good glucose consumption and CDT effects of OMV‐DFA inspired us to further conduct a comprehensive evaluation of its in vivo therapeutic potential. After the indicated treatments, we measured the tumor volumes, tumor weights, and survival rates of the 4T1 tumor‐bearing mice. Compared with the PBS control group, the growth of tumors in mice receiving OMV‐Da treatment was slightly inhibited, with a tumor growth inhibition (TGI) being ≈ 37% (Figure 4D–G), which might be primarily due to the immune effect of OMV in the tumor. Although tumor growth inhibition was enhanced by OMV‐DaFe (TGI ≈ 60%) and OMV‐DaAu (TGI ≈ 66%), all mice died within 40 and 44 days, respectively (Figure 4H). In sharp contrast, tumor growth almost stopped after OMV‐DFA treatment, and the survival rate of tumor‐bearing mice remained at 83.3% at the defined endpoint (day 50). Such a disparity in therapeutic effect was further confirmed by Ki67 and TUNEL staining of tumor sections (Figure S9, Supporting Information), supporting the superior therapeutic properties of OMV‐DFA.

### 2.5 Immunomodulatory Effects of OMV‐DFA for Tumor Inhibition

**Figure 5:**
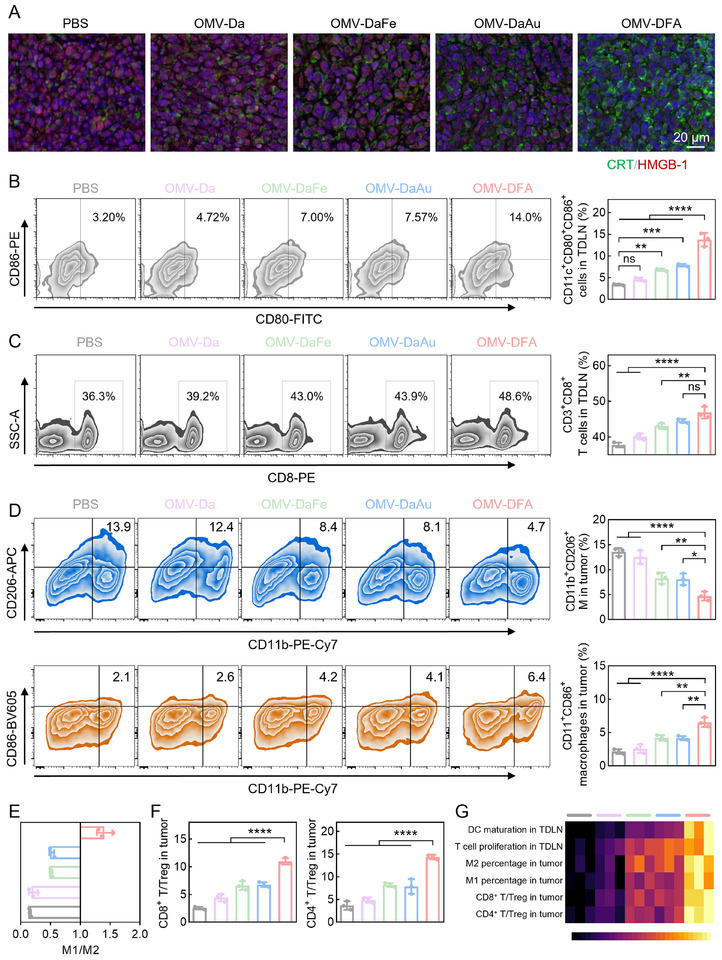
Evaluation of the immunomodulatory effects induced by OMV‐DFA. A) Immunofluorescence staining analysis of CRT and HMGB1 in tumor tissues following different treatments; nuclei are stained blue with DAPI. B) Representative flow cytometry plots and corresponding quantitative analysis of mature DCs (gated on CD11c⁺CD80⁺CD86⁺) in TDLNs. C) Representative flow cytometry plots and corresponding quantitative analysis of cytotoxic CD8⁺ T cells (gated on CD3⁺CD8⁺) in TDLNs. D) Representative flow cytometry plots and corresponding quantitative analysis of M1‐like (upper) and M2‐like (lower) macrophages in tumor tissues. E) M1/M2 macrophage ratio in tumor tissues. F) Ratios of CD8⁺ T cells (CD3⁺CD8⁺) to Tregs (CD4⁺CD25⁺Foxp3⁺) and CD4⁺ T cells (CD3⁺CD4⁺) to Tregs in tumor tissues. G) Summary of immune activation effects induced by OMV‐DFA treatment. Data in B‐F are presented as the mean ± SD (*n* = 3). Statistical significance was assessed using one‐way ANOVA. ^*^ *p* < 0.05, ^**^ *p* < 0.01, ^***^ *p* < 0.001, ^****^ *p* < 0.0001, ns: not significant.

Given that the in vitro ICD of 4T1 cells was induced by OMV‐DFA, we next evaluated the immunological responses triggered by OMV‐DFA. To this end, we first detected CRT expression and HMGB1 release in tumors, given their utility as ICD indicators. As expected, both CRT expression and HMGB1 release increased in the order of PBS, OMV‐Da, OMV‐DaFe, OMV‐DaAu, and OMV‐DFA, suggesting the potent ICD‐inducing ability of OMV‐DFA in vivo (**Figure** **5**A). Subsequently, we conducted a detailed investigation in the tumor‐draining lymph nodes (TDLNs), which lie immediately downstream of tumors and can be remodeled from immunosuppressive to immunostimulatory for anticancer immunotherapy. As shown in Figure 5B, OMV‐DFA treatment induced a DC maturation rate of 13.8% whereas the rate in the other groups was below 8.0%. Correspondingly, OMV‐DFA treatment also triggered the highest CD8^+^ cytotoxic T lymphocyte (CTL) proliferation among all the investigated groups (Figure 5C; Figure S10, Supporting Information).

Considering the potential of OMVs in tumor immune microenvironment reprograming, we analyzed the tumor immune microenvironment following different treatments. As expected, the OMV‐DFA group exhibited a clear reversal in the ratio of M1‐polarized TAMs to M2‐polarized TAMs (M1/M2 > 1) (Figure 5D,E). This shift toward an M1‐dominant phenotype was crucial, as M1 macrophages were known to secrete pro‐inflammatory cytokines (e.g., IL‐12, TNF‐α) and enhance antigen presentation, thereby promoting anti‐tumor immunity, whereas M2 macrophages typically supported tumor progression and immune suppression. We speculated that this polarization of tumor macrophages to the M1 phenotype could be explained by the synergism of the potent ICD effect of tumor cells induced by the OMV‐DFA shell and the microenvironment reprograming ability of OMVs. The pH‐responsive release of Fe ions and AuNPs from OMV‐DFA likely contributed to additional metabolic and oxidative stress within the tumor, further suppressing M2 polarization and reinforcing a pro‐inflammatory immune landscape.

Furthermore, OMV‐DFA treatment led to a significant increase in the ratio of both CD8^+^ effector T cells to Treg cells and CD4^+^ effector T cells to Treg cells, and more tumor cell death in tumors (Figure 5F,G). Collectively, OMV‐DFA effectively triggered potent immunological responses after administration. This shift was indicative of a more immunostimulatory TME, where the suppression exerted by Tregs was diminished, allowing for enhanced cytotoxic T lymphocyte (CTL) function. Given that ICD can promote dendritic cell (DC) maturation and antigen presentation, the increased infiltration and activation of effector T cells may be attributed to enhanced priming by antigen‐presenting cells (APCs) in response to tumor antigen release. Additionally, the immunogenic properties of OMVs, which contain pathogen‐associated molecular patterns (PAMPs) capable of activating pattern recognition receptors (PRRs) on APCs, likely contribute to the observed increase in T cell‐mediated tumor clearance. Collectively, these results demonstrate that OMV‐DFA not only induced potent ICD but also effectively reprogramed the tumor immune microenvironment by promoting M1 macrophage polarization, enhancing effector T cell infiltration, and reducing Treg‐mediated immunosuppression. These immunomodulatory effects synergistically contribute to improved anti‐tumor immune responses, ultimately leading to increased tumor cell death.

### 2.6 Inhibition of Tumor Recurrence and Tumor Metastasis by OMV‐DFA

**Figure 6:**
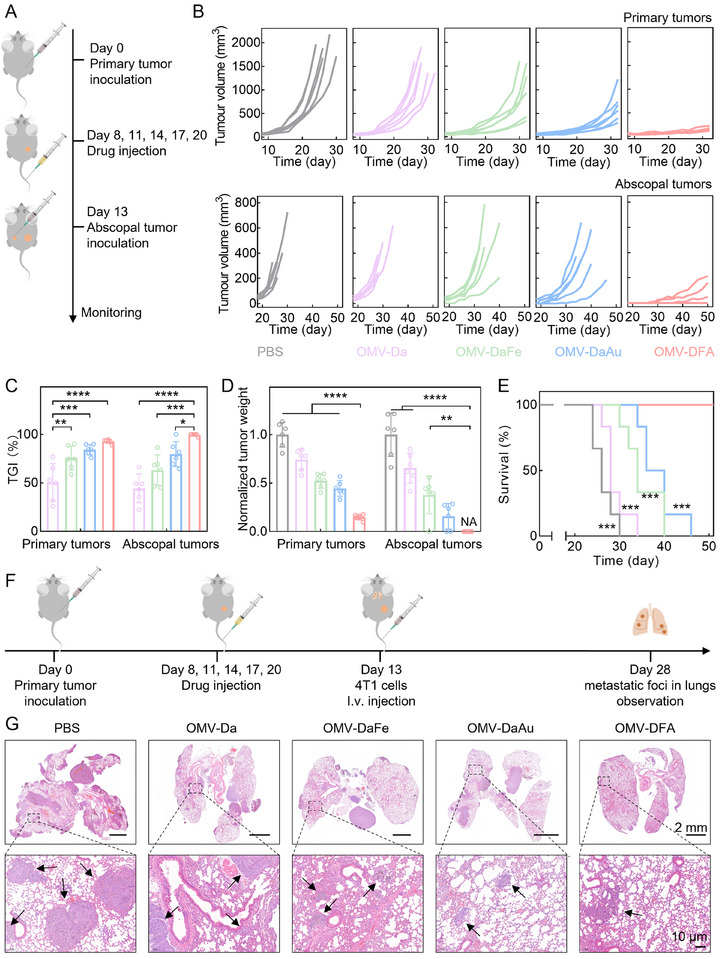
OMV‐DFA‐mediated inhibition of tumor recurrence and metastasis in a 4T1 tumor model. A) Schematic representation of the experimental design for the tumor recurrence model. B) Growth kinetics of primary and abscopal tumors in tumor‐beating mice treated with PBS, OMV‐Da, OMV‐DaFe, OMV‐DaAu, or OMV‐DFA. (*n* = 6). C) TGI rates at day 22 following different treatments. D) Weights of primary and rechallenged tumors at day 22 for each treatment group. E) Kaplan‐Meier survival curves of mice treated with the indicated formulations. (*n* = 6). F) Schematic representation of the experimental design to evaluate the inhibition of tumor metastasis by OMV‐DFA. G) Representative H&E‐stained lung tissue sections were analyzed for metastatic foci. Black arrows indicate regions of metastatic foci in lung tissues. Data in C and D are presented as the mean ± SD (*n* = 6). Statistical significance was assessed using one‐way ANOVA (C and D) and Log‐rank tests (E). ^*^ *p* < 0.05, ^**^ *p* < 0.01, ^***^ *p* < 0.001, ^****^ *p* < 0.0001.

To further explore the potential effects of the OMV‐DFA‐triggered immunological response on physically distant tumors, we continued to use a dual‐tumor model, wherein the second tumor was inoculated at the offside flank after the indicated treatment (**Figure** **6**A). We found that OMV‐DaFe and OMV‐DaAu only induced a moderate inhibitory effect on tumor development at both primary and distant tumor sites (Figure 6B–D). Correspondingly, the mice in these two groups died rapidly within 46 days (Figure 6E). In contrast, both primary and distant tumors were inhibited, with a TGI over 92%, and all mice remained alive at the defined endpoint.

To mimic the more advanced metastatic process, we established a hematogenous metastasis model for further investigation. Typically, mice xenografted with 4T1 tumors received the indicated treatments, followed by another intravenous injection of 4T1 cells (Figure 6F). Metastatic nodules in the lungs were detected using hematoxylin and eosin (H&E) staining. As shown in Figure 6G and Figure S11 (Supporting Information), much less metastatic nodules were observed in the OMV‐DFA group compared to the other groups, indicating the significant potential of the OMV‐DFA‐triggered immunological response for preventing metastasis. Additionally, no significant abnormalities were observed in body temperature, inflammatory cytokines, and H&E staining of main organs of mice after treatments with OMV‐DFA. The levels of blood urea nitrogen (BUN), lactate dehydrogenase (LDH), alanine aminotransferase (ALT), aspartate transaminase (AST), and alkaline phosphatase (ALP) following OMV‐DFA treatments were also within the normal range, which collectively confirmed the safety of OMV‐DFA for tumor inhibition (Figure S12, Supporting Information).

## 3 Conclusion

In this study, we developed an innovative OMVs‐based nanoparticle platform for tumor therapy by coating bacterial outer membrane vesicles (OMVs) with DaFe shells and incorporating ultrasmall Au nanoparticles via in situ growth method. This design leverages the DaFe network to effectively camouflage OMVs, mitigating systemic toxicity while enhancing tumor accumulation. The DaFe shell exhibits pH‐sensitive depolymerization in the acidic tumor microenvironment, leading to the controlled release of Fe ions, Au nanoparticles, and OMVs, thereby ensuring their activity is maximized at the tumor site while minimizing off‐target effects. The liberated Au nanozymes catalyze glucose oxidation into gluconic acid and hydrogen peroxide (H₂O₂), inducing starvation therapy (ST) while generating H₂O₂ to further accelerate Fe ion reaction. These Fe ions then participate in Fenton reactions with H₂O₂ to produce hydroxyl radicals (•OH), triggering chemodynamic therapy (CDT) and inducing ferroptosis and immunogenic cell death (ICD). This process promotes the release of tumor‐associated antigens, which, combined with the immunostimulatory properties of OMVs, trigger robust immune activation. The concurrent release of OMVs amplifies this response by activating antigen‐presenting cells (APCs), enhancing dendritic cell maturation, and promoting effector T cell infiltration. This cascade of events effectively alleviates immune suppression within the tumor microenvironment (TME) and facilitates tumor destruction. The ternary cascade mechanism, integrating AuNP‐mediated ST, Fe ion‐mediated CDT, and OMV‐induced immune activation, synergistically reprograms the TME, increasing M1 macrophage polarization, enhancing CD8⁺ and CD4⁺ T cell infiltration, and reducing regulatory T cell (Treg) populations. These effects collectively contribute to the inhibition of tumor progression, metastasis, and recurrence. Importantly, OMVs, Fe ions, and ultrasmall Au nanoparticles were metabolizable, ensuring a favorable safety profile. This study provides critical theoretical and methodological advancements in the utilization of modified OMVs as scaffolds for immune activation and tumor therapy. The proposed approach demonstrates strong potential for treating metastatic and recurrent tumors, laying a solid foundation for further refinement of tumor therapeutic strategies and improving cancer treatment outcomes.

## Conflict of Interest

The authors declare no conflict of interest.

## Supporting information



Supporting Information

## Data Availability

The data that support the findings of this study are available from the corresponding author upon reasonable request.
