# Supplementary material for: Chimeric Nanozyme Bacterial Outer Membrane Vesicles Reprograming Tumor Microenvironment for Safe and Efficient Anticancer Therapy
Source: Adv Sci (Weinh). 2025 Apr 25;12(22):2417712. doi: 10.1002/advs.202417712 (PMC12165108; doi:10.1002/advs.202417712)
Supplement: Supplementary file 1 — Supporting Information [file ADVS-12-2417712-s001.docx]

Supporting Information

**Chimeric Nanozyme Bacterial Outer Membrane Vesicles Reprograming Tumor Microenvironment for Safe and Efficient Anticancer Therapy**

Fan Zhang^#^, Qianqian Li^#^, Haibing Dai^#^, Weiqun Li*, Xiang Chen, Huibin Wu, Shanming Lu, Ran Luo, Feng Li, Guihong Lu*, Jianbo Yu*, Lin Mei*

G. H. Lu

Center for Child Care and Mental Health (CCCMH), Shenzhen Children's Hospital, Shenzhen, Guangdong 518038, China

E-mail: luguihong@ipe.ac.cn (G. H. Lu)

F. Zhang, H. B. Dai, W. Q. Li, X. Chen, H. B. Wu, S. M. Lu, J. B. Yu

Longgang Central Hospital, Shenzhen, Guangdong 518100, China

E-mail: szlcyxy@gzucm.edu.cn (J. B. Yu); liweiqun@hit.edu.cn (W. Q. Li)

L. Mei, F. Zhang, R. Luo,

State Key Laboratory of Advanced Medical Materials and Devices, Tianjin Key Laboratory of Biomedical Materials, Key Laboratory of Biomaterials and Nanotechnology for Cancer Immunotherapy, Institute of Biomedical Engineering, Chinese Academy of Medical Sciences and Peking Union Medical College, Tianjin 300192, China

E-mail: meilin@bme.pumc.edu.cn (L. Mei)

Q. Q. Li

Institute of Pharmaceutics, Shenzhen Campus of SunYat-sen University, Shenzhen, Guangdong 518107, China

Shenzhen Bay Laboratory, Shenzhen, Guangdong 518132, China

F. Li

Key Laboratory of Biopharmaceutical Preparation and Delivery, Chinese Academy of Sciences, Beijing, 100190, China.

# F. Zhang, Q. Q. Li, H. B. Dai contributed equally to this work

1. Materials and Methods

**1. 1 Materials and Reagents**

RPMI 1640 medium, Dulbecco's Modified Eagle Medium (DMEM), and fetal bovine serum (FBS) were purchased from Gibco Life Technologies (AG, Switzerland). Trypsin-EDTA (0.25%) and penicillin-streptomycin were obtained from Corning Life Sciences (Wujiang, China). The Micro BCA Protein Assay Kit and Calcein-AM/PI Live/Dead Detection Kit were purchased from Thermo Fisher (USA). The Cell Counting Kit-8 (CCK-8), 2′,7′-dichlorofluorescin diacetate (DCFH-DA), and ELISA kits were obtained from Solarbio Life Sciences (Beijing, China). Hoechst 33342 was supplied by Life Technologies (USA). Anti-mouse HMGB-1 and anti-mouse CRT antibodies were sourced from Abcam (UK). FITC-conjugated anti-mouse CD11c antibody was from Proteintech (China), while APC-conjugated anti-mouse CD11b, APC-conjugated anti-mouse CD80, PE-conjugated anti-mouse CD86, APC-conjugated anti-mouse CD3, FITC-conjugated anti-mouse CD8, FITC-conjugated anti-mouse CD4, PE-conjugated anti-mouse CD25, PacificBlue-conjugated anti-mouse Foxp3, and PE-conjugated anti-mouse IFN-γ antibodies were obtained from BioLegend (USA). Escherichia coli (BL21) was purchased from the China General Microbiological Culture Collection Center (CGMCC). Sodium chloride, sodium hydroxide, and other chemicals were sourced from Aladdin.

1.2 Cell lines.

4T1 murine mammary carcinoma cells, and 4T1-luc murine mammary carcinoma cells were obtained from China Infrastructure of Cell Line Resource, and they were grown in RPMI 1640 Medium supplemented with FBS (10%) and penicillin-streptomycin (1%). Cells were kept in a humidified atmosphere with 5% CO_2_.

1.3 Animals.

Balb/c mice were obtained from Beijing Vital River Laboratory (Beijing, China). They are all female, 6-8 weeks, being raised in rooms with controlled conditions (23 °C, 55 ± 5% humidity, and 12 hours of light-12 hours of darkness). Animal experiments were conducted according to the Guide for the Care and Use of Laboratory Animals (China, GB/T 35892-2018), and received approval from Ethics Review Committec of Longgang Central Hospital on Laboratory Animal Care (No.2024-059)

**1.4 Isolation of Bacterial Outer Membrane Vesicles (OMVs)**

*Escherichia coli* was cultured overnight in LB medium on a rotary shaker (37 °C) to achieve high cell densities. The OMVs-containing supernatant was separated by centrifugation (8,000 g, 4 °C, 20 minutes) followed by filtration through a 0.45-μm membrane. The supernatant was then concentrated using membrane filtration and subjected to ultracentrifugation (150,000 g, 3 hours, 4 °C), resulting in OMVs with an approximate particle size of 50 nm. The OMVs protein concentration was determined using the Micro BCA Protein Assay Kit.

**1.5 Preparation of OMV-DFA**

A one-pot method was used to prepare OMV-DFA. OMVs (50 µL, 30 mg/mL) were mixed with HEPES saline buffer (2 mL, 50 mM) supplemented with 10 mM Fe(NH₄)₂·(SO₄)₂·6H₂O, followed by the addition of 0.01 mM 5-hydroxydopamine modified PEG-SH (SH-PEG-Dopamine). The mixture was rapidly introduced into 1 mM Tris-HCl buffer containing 10 mM HAuCl₄ and reacted with 0.1 mM NaBH₄ for 5 minutes. After incubation, OMV-DFA was collected by centrifugation (15,000 g for 15 minutes) and washed three times with ultrapure water.

**1.6 Characterization of OMVs and OMV-DFA**

Transmission electron microscopy (TEM) (JEM-1400 system, Japan) was used to examine the morphology and particle size of OMVs and OMV-DFA. Elemental mapping and energy dispersive spectrometry (EDS) analysis were obtained using a TEM equipped with an EDS attachment. Zeta potential and hydrodynamic diameter of OMVs and OMV-DFA were assessed by a Zeta Sizer (Nano series, Malvern, UK). The absorbance spectra of OMV-DFA were recorded using a multifunctional microplate reader (TECAN, Switzerland).

To assess glucose consumption activities, OMV@DaFe or OMV-DFA (100 μg protein/mL) were dispersed in 2 mL glucose solution (0.01 M). The glucose and gluconic acid contents were detected by a glucose kit (Solarbio, China). To assess Fe ions ralease behavior, OMV-DFA (100 μg protein/mL) was dispersed in 2 mL PBS (pH 7.4 or 6.2). The Fe ions concentration were detected using corresponding detection kit based o-phenanthroline chromogenic reaction at the indicated time-points. To assess •OH producing ability, OMV-DFA (100 μg protein/mL) was dispersed in 200 μL PBS with or without 200 μM H_2_O_2_. The •OH radicals were detected using a methylene blue kit (Solarbio, China) at the indicated time-points or using an electron spin resonance (ESR) spectrometer (Bruker E500 spectrometer, Bruker Biospin, GER).

Proteomic analysis. Proteins were isolated from frozen OMV and OMV-DFA using SDT lysis method and quantified by BCA assay kit. Then the peptides were made by enzymolysis with filter-aided proteome preparation and desalination with C18 Cartridge. The mass spectrometry was conducted using Q Exactive™ Hybrid Quadrupole-Orbitrap™ Mass Spectrometer (Thermo Fisher Scientific). The raw MS files were analyzed and searched against target protein database based on the species of the samples using Proteome Discoverer 2.5. Both LC-MS/MS analysis and Data analysis were done by Beijing Biotech-Pack Scientific Co. Ltd (China).

1.7 *In vitro* cytotoxicity studies

4T1 cells were placed into 96-well cell culture plates (10⁴/well) and incubated overnight at 37 °C. Then the culture medium was replaced with new RPMI 1640 medium supplemented with varying concentrations of OMV-DFA (Protein: 0 to 200 μg/mL). Cell viability was evaluated using a CCK-8 assay after an additional 24 hours of culture.

According to above experimental result, 4T1 cells were treated with PBS, OMV-Da, OMV-DaAu, OMV-DaFe, or OMV-DFA (200 μg protein/mL) under the same culture conditions. Cell viability was evaluated using a CCK-8 assay after 24 hours of culture.

For Live/Dead detection, 4T1 cells were incubated with different treatments (200 μg protein/mL) for 24 hours. Cells were stained using calcein-AM to visualize living cells and PI to characterize dead cells. All experimental procedures were carried out following the manufacturer’s directions.

To assess the effect of glucose consumption and ROS produce abilities *in vitro*, cultured 4T1 cells were added PBS, OMV-Da, OMV-DaFe, OMV-DaAu, or OMV-DFA (100 μg protein/mL). After culturing at 37 °C for 12 h, glucose consumption and ROS generation in the cultures were detected using a glucose uptake and transport probe (2-NBDG) or free radicals fluorogenic probes (DCFH-DA or hydroxyphenyl fluorescein) by confocal laser scanning microscopy (CLSM) (Leica, Germany) and flow cytometry (Beckman Coulter, CytoFLEX).

1.8 Evaluation of ICD *in vitro*

To analyze CRT expression and intracellular HMGB1 distribution *in vitro*, 4T1 cells were incubated with PBS, OMV-Da, OMV-DaAu, OMV-DaFe, or OMV-DFA (100 μg protein/mL) for 24 hours of incubation. After that, cells were performed PBS rinse, 15 min-fixation in 4% paraformaldehyde, and 10 min-permeabilization using 0.25% Triton X-100, blocking with 1% (w/v) BSA, 12 h-incubation with anti-CRT or anti-HMGB1 antibodies at 4 °C, 30 min-staining with fluorescent secondary antibodies, and finally Hoechst 33342 staining. Confocal images were taken to observe the cells.

To evaluate DC maturation and macrophage polarization *in vitro*, 4T1 cancer cells were seeded in 48-well plates at 2×10^4^ cells/well. 12 h later, these cells were divided into five groups and incubated with PBS, OMV-Da, OMV-DaAu, OMV-DaFe, or OMV-DFA, respectively, in which the final OMV concentration was 100 μg/mL for the last four groups. After 24-h incubation, the supernatants were collected and added into the DCs and macrophages cultured in 24-well plates. After 48 h, these DCs and macrophages were analyzed using flowcytometry to detect the expression of DC maturation markers, including CD80 and CD86, and macrophage polarization markers, including CD86 and CD206.

**1.9 Tumor accumulation ability of OMV-DFA**

Balb/c mice were subcutaneously administered 4T1 cells (2 × 10^5^ cells/mouse). When tumor volumes attained 150 mm³ at day 14, mice were intravenously injected with OMVs or OMV-DFA (0.5 mg protein/kg) at day 14 and Cyanine7 (Cy7)-labeled OMVs or OMV-DFA (0.5 mg protein/kg) at day 15. Fluorescence imaging proceeded at various time intervals. For ex vivo imaging, mice were euthanized 24 hours after injection, and their major organs (heart and liver, etc) and tumors were harvested for visualization. For histological evaluation, 4T1 tumor-containing mice were injected with Cy3-labeled OMVs or OMV-DFA (0.5 mg protein/kg) at the second injection. The organs were frozen with OCT on dry ice and sliced (10-μm-thick) for observation. The DAPI staining images were captured with an automatic multispectral imaging approach.

**1.10 Evaluations of anti-tumor effect**

Balb/c mice were subcutaneously administered 4T1 cells (2 × 10^5^ cells/mouse). At day 8, tumor-bearing mice were randomly separated into five groups. Each group received intravenous injections of PBS, OMV-Da, OMV-DaAu, OMV-DaFe, or OMV-DF (0.5 mg protein/kg) at day 8, 11, 14, 17, 20. Tumor size will be measured at regular intervals to plot a tumor growth curve. Tumor volume was computed using the formula: (width)² × (length) × 1/2. Animals were sacrificed when their tumor volumes surpassed 1,000 mm³, and a survival curve will be generated. Tumor tissues collected from treated mice were sectioned and stained using a glucose kit and an Fe-induced cell death detection kit.

Tumors were fixed (4% paraformaldehyde), sliced (10 μm), and stained using Ki67 and TUNEL kits. They were visualized using an automated multispectral imaging system (PerkinElmer Vectra II, USA).

1.11 *In vivo* immune activation assay.

Balb/c mice were subcutaneously administered 4T1 cells (2 × 10^5^ cells/mouse). When tumor volumes attained 100 mm³, mice were separated into five groups. Each group received intravenous injections of PBS, OMV-Da, OMV-DaAu, OMV-DaFe, or OMV-DFA (0.5 mg protein/kg) every 3 days for three injections. The mice were euthanized for immune cell analysis. Draining lymph nodes (DLNs) and tumor tissues underwent collection and homogenization into single-cell mixtures. For dendritic cell (DC) maturation analysis, cells from DLNs were stained using CD11c, CD80, CD86 antibodies. For cytotoxic T lymphocyte (CTL) analysis, DLNs were stained using CD8, CD3, and IFN-γ antibodies. For CD8^+^, CD4^+^ T, Treg cell analysis, tumor cells staining with CD4, CD3, CD8, CD25 or FOXP3 antibodies. Stained cells were rinsed using PBS and analyzed using flow cytometry.

To evaluate HMGB1 and CRT in the tumor tissues, Balb/c mice were subcutaneously administered 4T1 cells (2 × 10^5^ cells/mouse). When tumor volumes attained 100 mm³, mice were separated into five groups. Each group received intravenous injections of PBS, OMV-Da, OMV-DaAu, OMV-DaFe, or OMV-DFA (0.5 mg protein/kg) every 3 days for three injections. The mice were euthanized for immunogenic cell death analysis. The tumors of both groups were collected and frozen in optimum cutting temperature (OCT) tissue compound (Sakura, Tokyo, Japan) on dry ice and then sectioned into 10-μm slices. The obtained tumor slices were incubated with HMGB1 and CRT antibodies, followed by orderly staining with fluorescence secondary antibody and 4′,6-diamidino-2-phenylindole (DAPI) solution. Last, the slices were imaged with an automatic multispectral imaging system (PerkinElmer).

1.12 Evaluation of recurrence and metastasis of OMV-DFA

For recurrence model construction, Balb/c mice were injected with 4T1-luc cells (2 × 10^5^ cells/mouse). At day 8, tumor-bearing mice were randomly separated into five groups and intravenously administered PBS, OMV-Da, OMV-DaAu, OMV-DaFe, or OMV-DFA (0.5 mg protein/kg) at day 8, 11, 14, 17, 20. At day 13, the mice were rechallenged using subcutaneous injections of 4T1 tumor cells (2 × 10^5^ cells). Tumor growth was evaluated every two days.

For lung metastasis model construction, Balb/c mice were injected with 4T1-luc cells (2 × 10^5^ cells). At day 8, tumor-bearing mice were randomly separated into five groups and intravenously administered PBS, OMV-Da, OMV-DaAu, OMV-DaFe, or OMV-DFA (0.5 mg protein/kg) at day 8, 11, 14, 17, 20. At day 13, mice were *i.v.* injected with 4T1-luc cells (1 × 10⁵ cells). Mice were then euthanized at day 28, and the lung tissue sections were H&E stained for metastasis nodules assessing.

1.13 Systemic toxicity evaluation

Balb/c mice were administered PBS (Healthy control group) or OMV-DFA (0.5 mg protein/kg) *via* intravenous injection every three days over three injections. Serum extracts were obtained at various time points to measure cytokine levels (TNF-α, IFN-γ, and IL-6) utilizing ELISA kits. The body temperature of the mouse was also recorded at different time points. The serum biochemistry levels of lactate dehydrogenase (LDH), alanine aminotransferase (ALT), aspartate transaminase (AST), alkaline phosphatase (ALP), and blood urea nitrogen (BUN) were evaluated with a biochemical autoanalyzer (TBA-40, Toshiba) at day 14 and 30. The major organs (heart, spleen, liver, lung, and kidneys) were obtained at day 14 and 30, and the degree of damage was assessed through H&E staining.

**1.14 Statistical analysis**

Statistical analysis of the data was performed using with GraphPad Prism 8.0.1 software by two-tailed unpaired Student’s t-tests, log-rank test, or one-way ANOVA. All results are expressed as mean ± standard error, unless otherwise noted. Statistical significance levels were set at * p <0.05, ** p < 0.01, *** p< 0.005, **** p < 0.001, ns: not significant.


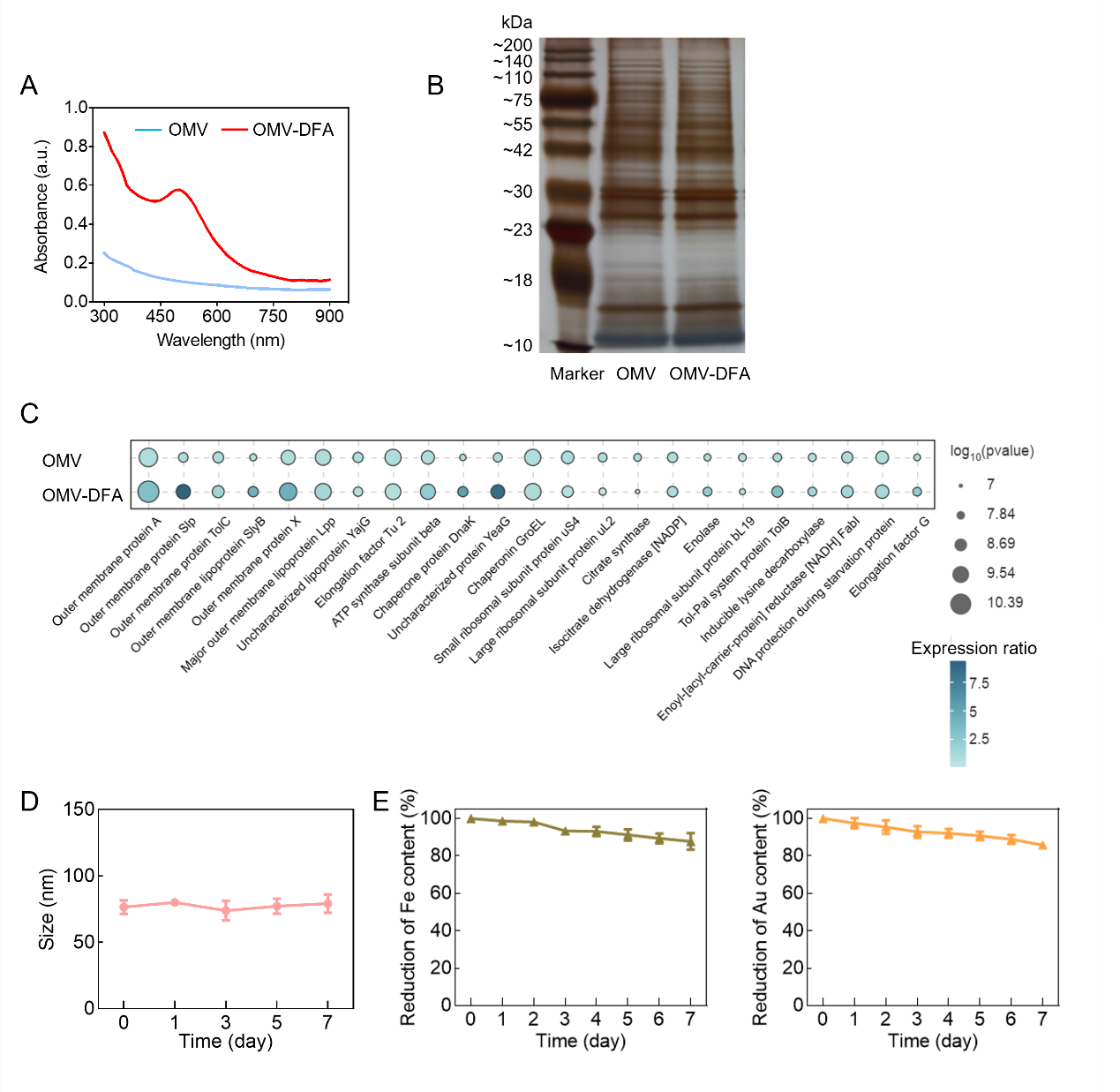


**Figure S1. Construction and characterization of OMV-DFA.**

1. UV-vis-NIR absorption spectra of OMVs and OMV-DFA.
2. SDS-PAGE analysis of OMVs and OMV-DFA.
3. Intensities comparation of representative proteins in OMVs and OMV-DFA obtained from proteomics analysis.
4. Size changes of OMV-DFA during storage in PBS for 7 days.
5. Fe and Au content changes in OMV-DFA during storage in PBS for 7 days.

Data in D and E are presented as the mean ± SD (n = 3).


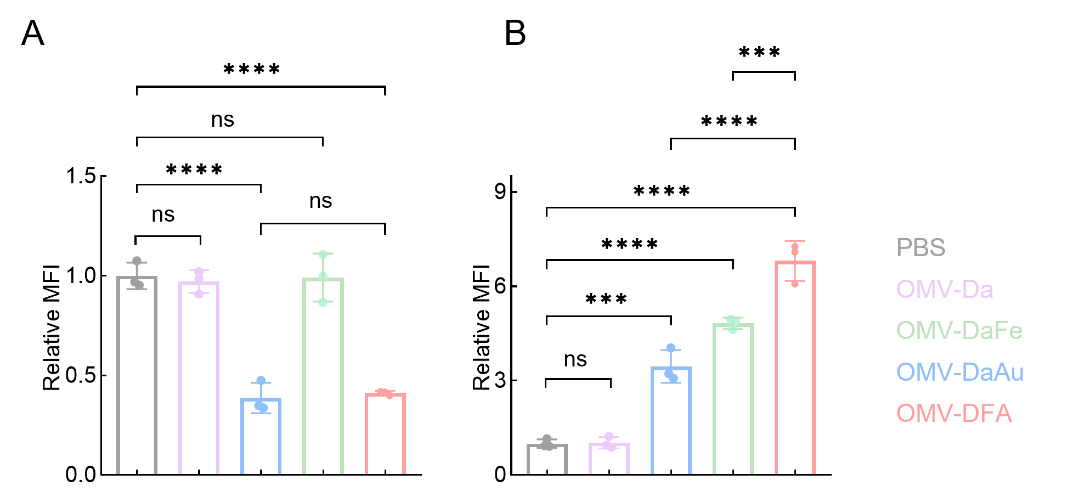


**Figure S2. Quantification of glucose fluorescent signal (A) and ROS fluorescent signal (B) in 4T1 cells**.

Data are presented as the mean ± SD (n = 3). Statistical significance was assessed using one-way ANOVA. ****p <* 0.001, *****p <* 0.0001, ns: not significant.


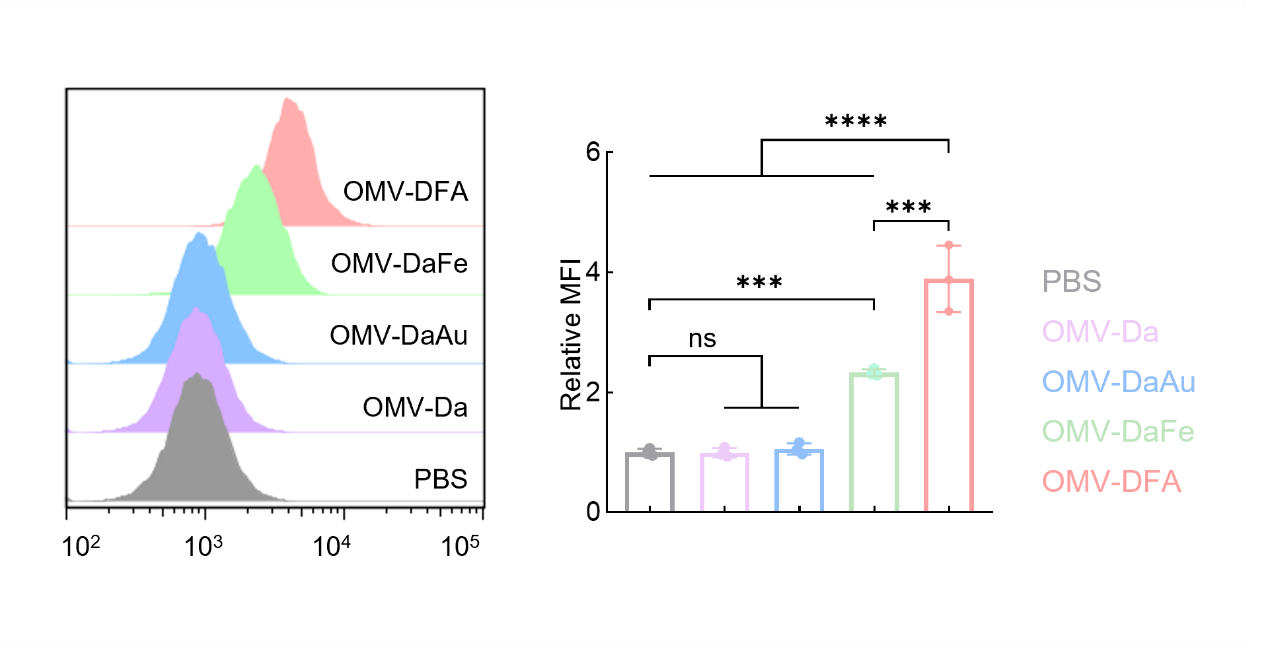


**Figure S3. Representative flowcytometry histograms and corresponding fluorescence intensity (FLI) of hydroxyphenyl fluorescein (HPF) for intracellular •OH detection after the indicated treatment.**

Quantification data are presented as the mean ± SD (n = 3). Statistical significance was assessed using one-way ANOVA. ***p < 0.001, ****p < 0.0001, ns: not significant.


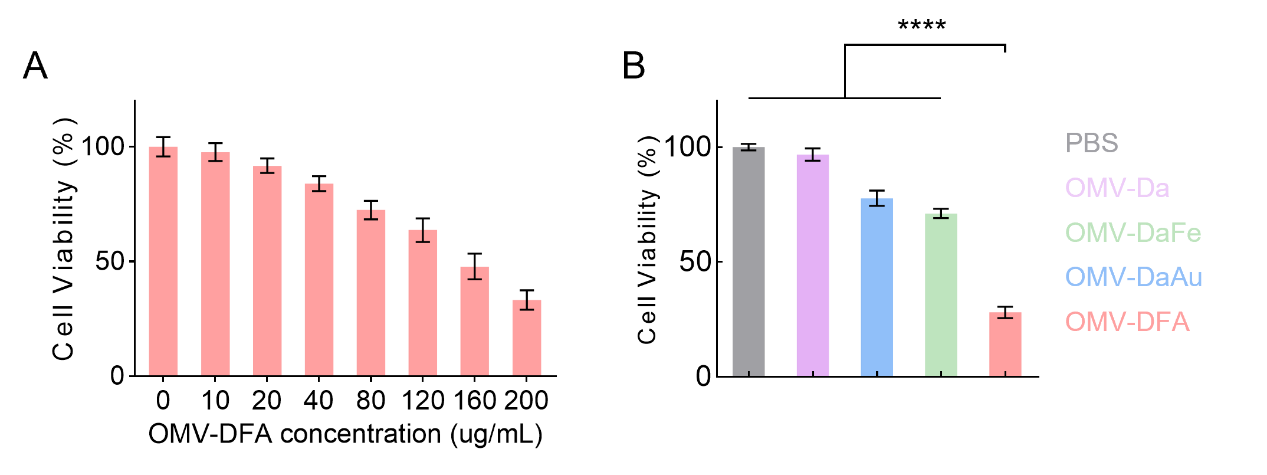


**Figure S4. Quantification of 4T1 cell viability after incubation with different concentrations of OMV-DFA (A) or different formulations (B)**.

Data are presented as the mean ± SD (n = 3). Statistical significance was assessed using one-way ANOVA. *****p <* 0.0001.


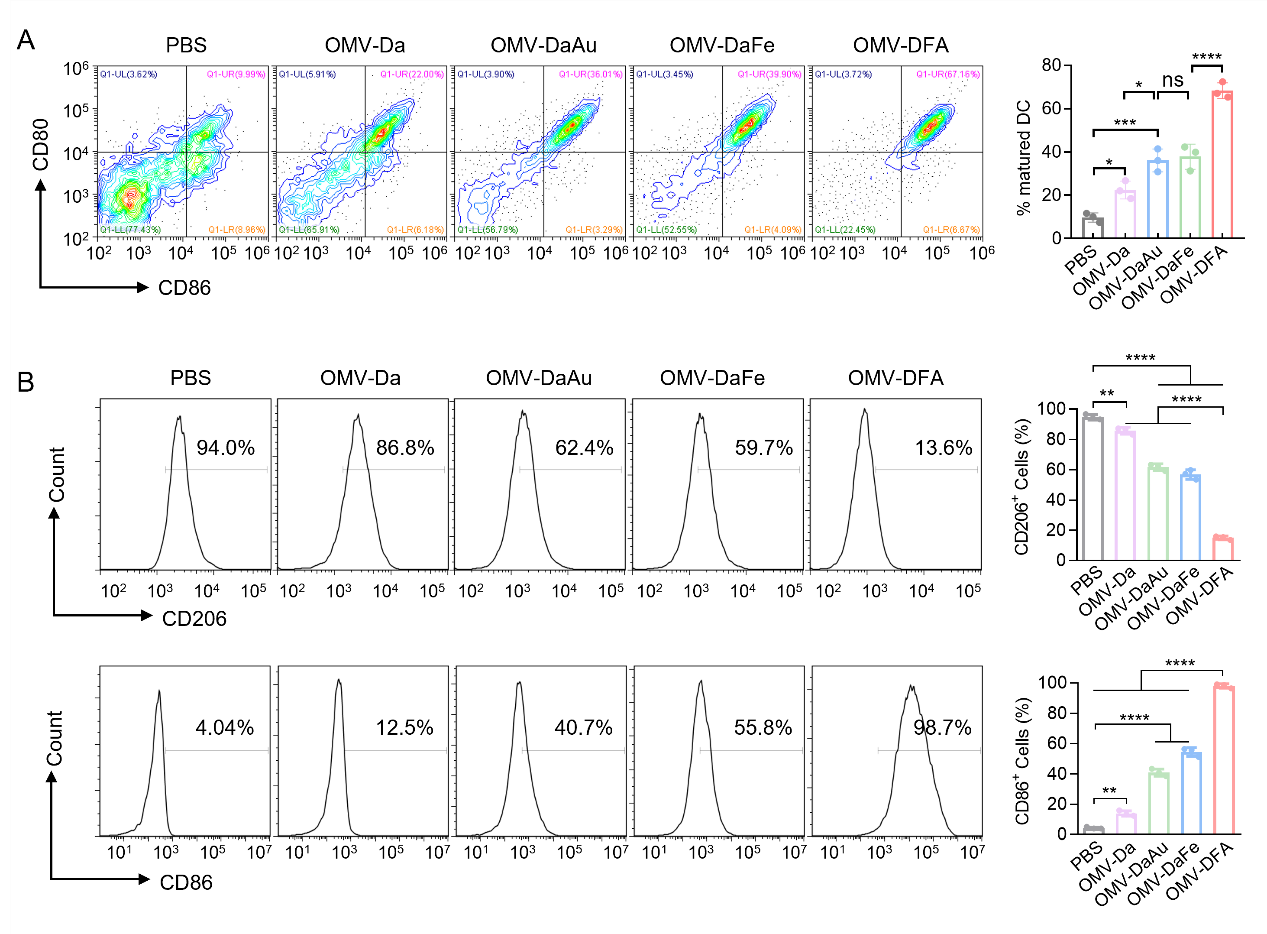


**Figure S5. In vitro evaluation of the ICD-induced DC maturation and macrophage polarization.** (A) Representative flowcytometry profiles showing DC maturation and quantification of ICD-induced DC maturation. (B) Representative flow cytometry plots and corresponding quantitative analysis of M2-like (upper) and M1-like (lower) macrophages after the indicated treatment.

Quantification data are presented as the mean ± SD (n = 3). Statistical significance was assessed using one-way ANOVA. **p <* 0.05, ***p <* 0.01, ****p <* 0.001, *****p <* 0.0001, ns: not significant.


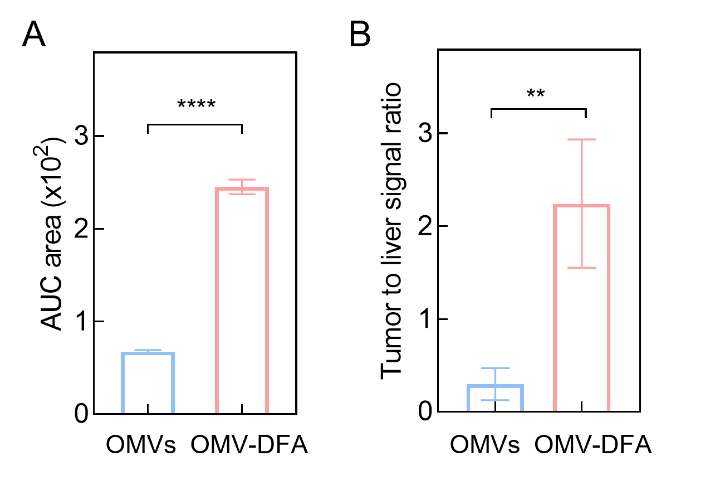


**Figure S6. (A) The area under the curve of Figure 3B. (B) Tumor-to-liver fluorescence signal ratio of different groups.**

Data are presented as the mean ± SD (n = 3). Statistical significance was assessed using two-tailed unpaired Student’s *t*-test. ***p <* 0.01, *****p <* 0.0001.


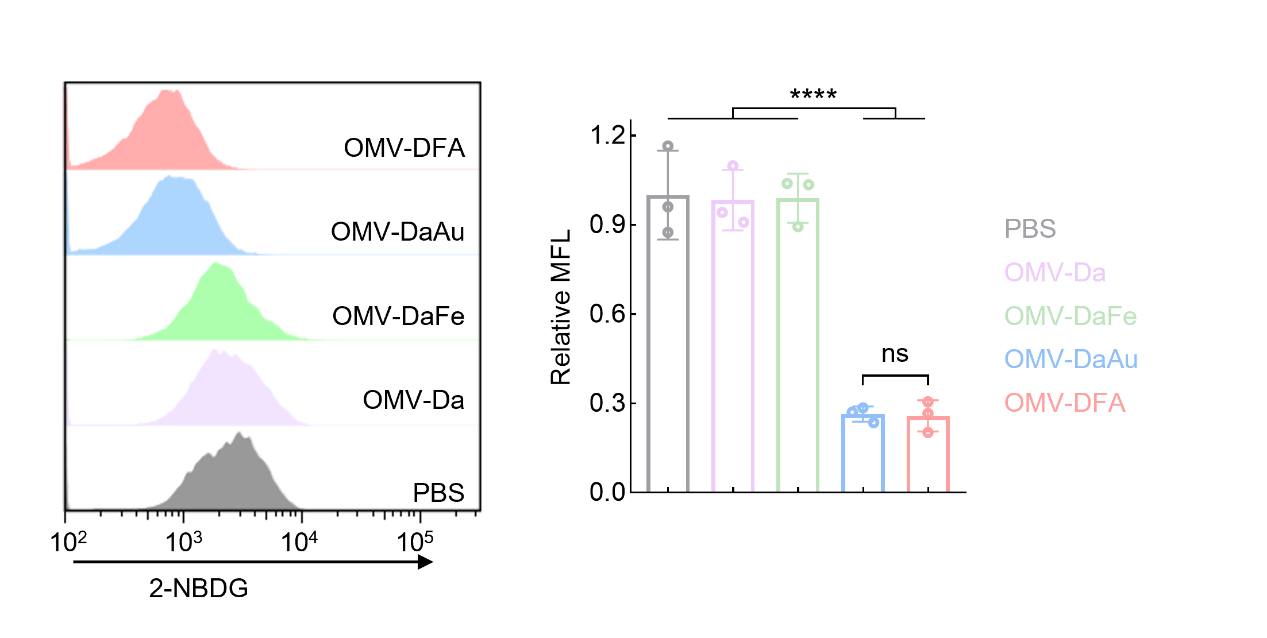


**Figure S7. Representative flowcytometry histogram of glucose in tumor (left) and corresponding quantification of fluorescent signal (right)**.

Data are presented as the mean ± SD (n = 3). Statistical significance was assessed using one-way ANOVA. *****p <* 0.0001, ns: not significant.


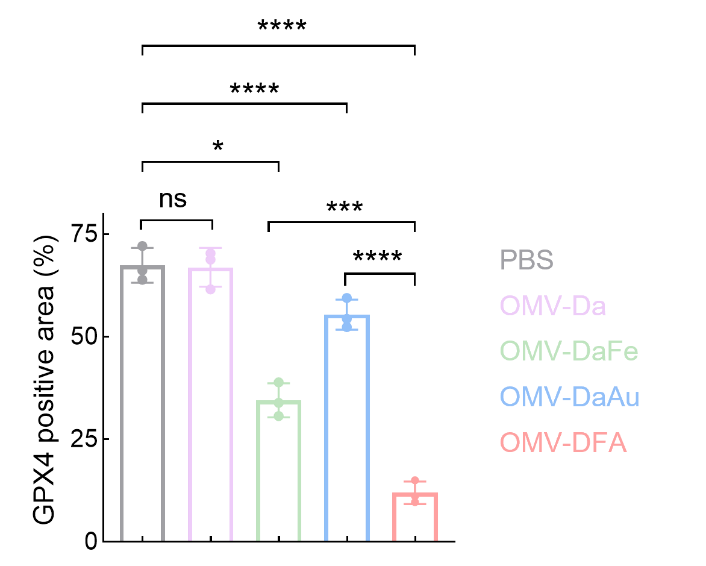


**Figure S8. Quantification of GPX4 positive area in tumor obtained from mice receiving the indicated treatments**.

Data are presented as the mean ± SD (n = 3). Statistical significance was assessed using one-way ANOVA. **p <* 0.05, ****p <* 0.001, *****p <* 0.0001, ns: not significant.


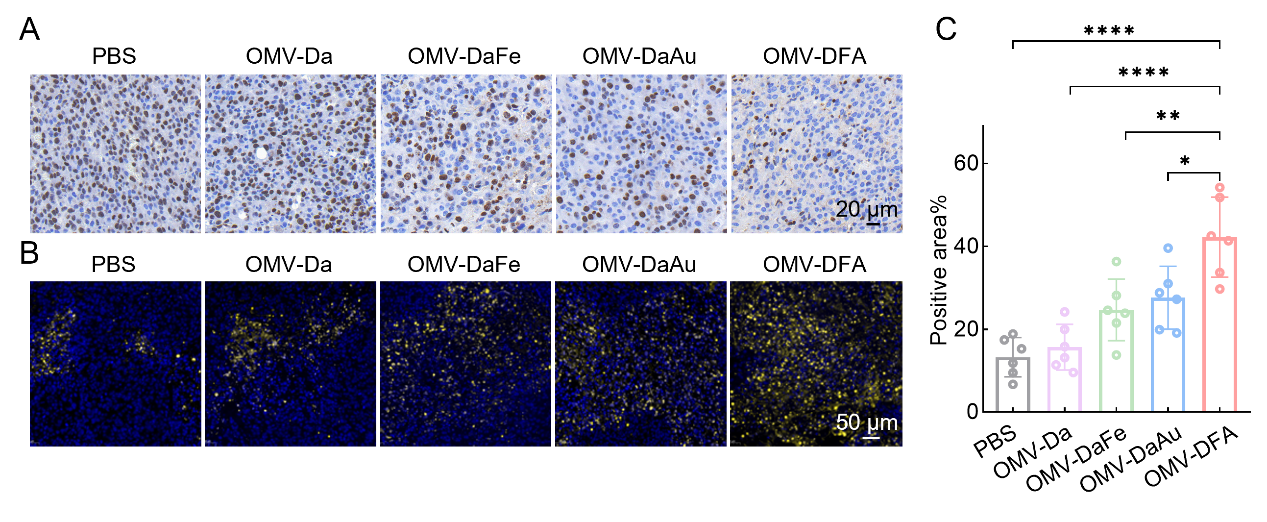


**Figure S9. Evaluation of the therapeutic effects using Ki67 and TUNEL.**

1. Immunohistochemical analysis for Ki67 expression in tumors obtained from mice receiving the indicated treatments.
2. TUNEL staining analysis for tumors after the indicated treatments.
3. Quantification of TUNEL positive area in tumor slices after the indicated treatments.

Data in C are presented as the mean ± SD (n = 6). Statistical significance was assessed using one-way ANOVA. **p <* 0.05, ****p <* 0.001, *****p <* 0.0001.


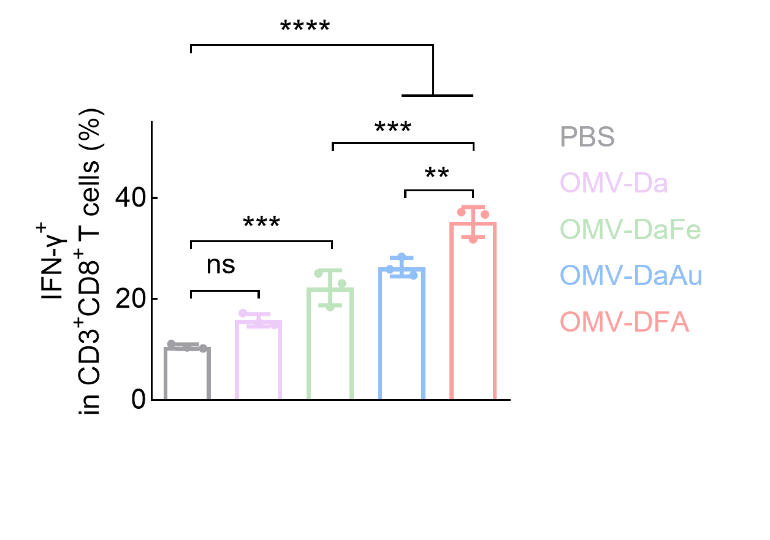


**Figure S10. Quantification of CTL percentage in CD8^+^ T cells in TDLN obtained from mice receiving the indicated treatments**.

Data are presented as the mean ± SD (n = 3). Statistical significance was assessed using one-way ANOVA. ****p <* 0.001, *****p <* 0.0001, ns: not significant.


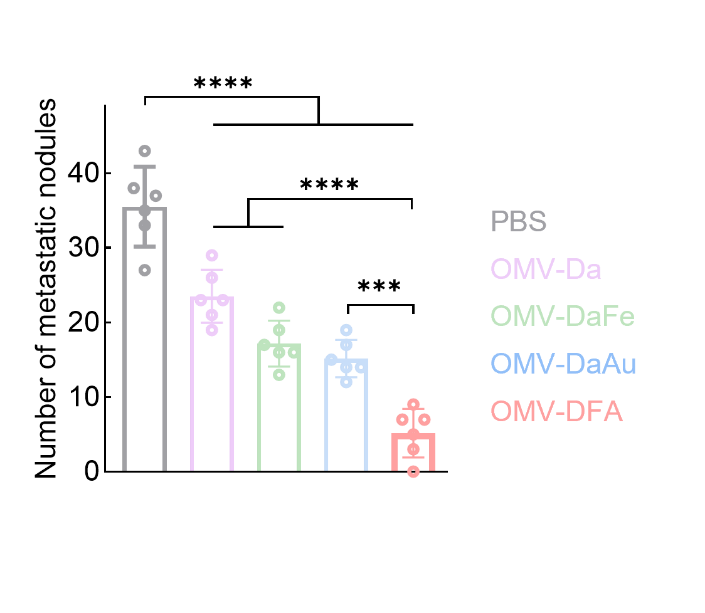


**Figure S11. Quantification of metastatic nodules in lungs obtained from mice receiving the indicated treatments**.

Data are presented as the mean ± SD (n = 6). Statistical significance was assessed using one-way ANOVA. ****p <* 0.001, *****p <* 0.0001.


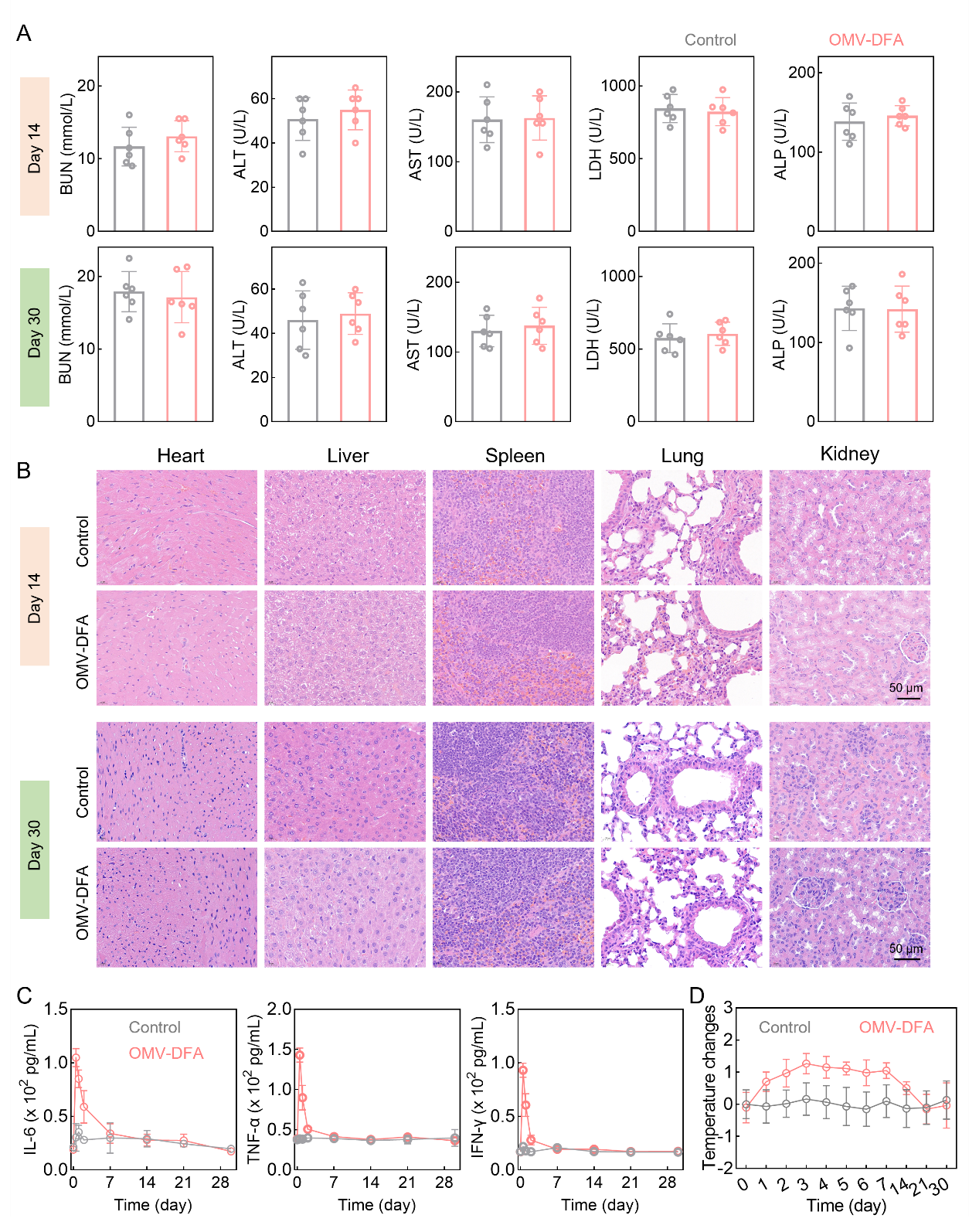


**Figure S12. Safety evaluation of OMV-DFA.**

1. Representative biochemical indices levels obtained from PBS and OMV-DFA-treated mice at day 14 and 30 (n = 6).
2. H&E staining analysis of major organs obtained from PBS and OMV-DFA-treated mice at day 14 and 30.
3. Systemic cytokine (IL-6, TNF-α, and IFN-γ) release profiles across varying durations (n = 3).
4. Body temperature changes during a 30-day monitoring period (n = 6).

Data are presented as the mean ± SD.

Table S1. The protein compositions in OMVs and OMV-DFA characterized using proteomics analysis.

| **Index** | **Accession** | **Protein Description** | **Score** | **Molecular Weight (kDa)** |
| --- | --- | --- | --- | --- |
| ***Extracellular region*** | |  |  |  |
| 1 | P06996;P21420 | Outer membrane porin C | 237.30 | 40.37 |
| 2 | P0A910 | Outer membrane protein A | 116.29 | 37.20 |
| ***Outer cell membrane*** | |  |  |  |
| 1 | P37194 | Outer membrane protein Slp | 84.32 | 20.96 |
| 2 | P02930 | Outer membrane protein TolC | 55.12 | 53.74 |
| 3 | P0A905 | Outer membrane lipoprotein SlyB | 45.41 | 15.60 |
| 4 | P0A917 | Outer membrane protein X | 40.30 | 18.60 |
| 5 | P69776 | Major outer membrane lipoprotein Lpp | 14.36 | 8.32 |
| 6 | P31554 | LPS-assembly protein LptD | 12.36 | 89.67 |
| ***Periplasm*** |  |  |  |  |
| 1 | P0A855 | Tol-Pal system protein TolB | 15.27 | 45.96 |
| 2 | P0AG82 | Phosphate-binding protein PstS | 13.81 | 37.02 |
| 3 | P64596 | Outer membrane lipoprotein DolP | 6.24 | 20.03 |
| ***Inner cell membrane*** | |  |  |  |
| 1 | P0CE48;P0CE47 | Elongation factor Tu 2 | 55.68 | 43.31 |
| 2 | P0ABB4 | ATP synthase subunit beta | 41.86 | 50.33 |
| 3 | P0A6Y8 | Chaperone protein DnaK | 11.48 | 69.11 |
| 4 | P0ADA5 | Uncharacterized lipoprotein YajG | 7.16 | 20.95 |
| ***Cytoplasm*** | |  |  |  |
| 1 | P0A6F5 | Chaperonin GroEL | 84.94 | 57.33 |
| 2 | P0A7V8 | Small ribosomal subunit protein uS4 | 32.58 | 23.47 |
| 3 | P0A717 | lipoproteins | 20.94 | 34.22 |
| 4 | P0A7Z4 | DNA-directed RNA polymerase subunit alpha | 20.22 | 36.51 |
| 5 | P0A9B2 | Glyceraldehyde-3-phosphate dehydrogenase A | 19.78 | 35.53 |
| 6 | P60422 | Large ribosomal subunit protein uL2 | 19.13 | 29.86 |
| 7 | P05055 | Polyribonucleotide nucleotidyltransferase | 17.76 | 77.10 |
| 8 | P0ABH7 | Citrate synthase | 15.73 | 48.01 |

| **Index** | **Accession** | **Protein Description** | **Score** | **Molecular Weight (kDa)** |
| --- | --- | --- | --- | --- |
| 9 | P08200 | Isocitrate dehydrogenase [NADP] | 15.34 | 45.76 |
| 10 | P0A8V2 | DNA-directed RNA polymerase subunit beta | 13.95 | 150.63 |
| 11 | P0A870 | Transaldolase B | 12.22 | 35.22 |
| 12 | P0A6M8 | Elongation factor G | 12.05 | 77.58 |
| 13 | P0A9H3 | Inducible lysine decarboxylase | 11.69 | 81.26 |
| 14 | P0A7W1 | Small ribosomal subunit protein uS5 | 10.38 | 17.60 |
| 15 | P06959 | Dihydrolipoyllysine-residue acetyltransferase component of pyruvate dehydrogenase complex | 10.36 | 66.10 |
| 16 | P0AEK4 | Enoyl-[acyl-carrier-protein] reductase [NADH] FabI | 8.18 | 27.86 |
| 17 | P0ABT2 | DNA protection during starvation protein | 7.69 | 18.70 |
| 18 | P0A6P9 | Enolase | 6.94 | 45.65 |
| 19 | P0A7K6 | Large ribosomal subunit protein bL19 | 6.70 | 13.13 |
| ***Unknown*** |  |  |  |  |
| 1 | P04128 | Type-1 fimbrial protein, A chain | 75.72 | 18.11 |
| 2 | P09169 | Protease 7 | 74.32 | 35.56 |
| 3 | P0ACY3 | Uncharacterized protein YeaG | 57.97 | 74.48 |
| 4 | P69908;P69910 | Glutamate decarboxylase alpha | 47.21 | 52.69 |
| 5 | P0A799 | Phosphoglycerate kinase | 33.59 | 41.12 |
| 6 | P0A8T7 | DNA-directed RNA polymerase subunit beta' | 31.60 | 155.16 |
| 7 | P0A991 | Fructose-bisphosphate aldolase class 1 | 26.53 | 38.11 |
| 8 | P0AG67 | Small ribosomal subunit protein bS1 | 19.51 | 61.16 |
| 9 | P33602 | NADH-quinone oxidoreductase subunit G | 18.35 | 100.30 |
| 10 | P0ABD3 | Bacterioferritin | 18.10 | 18.50 |
| 11 | P09373;P42632 | Formate acetyltransferase 1 | 17.82 | 85.36 |
| 12 | P33570;P27302 | Transketolase 2 | 17.46 | 73.04 |
| 13 | P00509 | Aspartate aminotransferase | 16.56 | 43.57 |

| Index | Accession | Protein Description | Score | Molecular Weight (kDa) |
| --- | --- | --- | --- | --- |
| 14 | P0DTT0 | Large ribosomal subunit assembly factor BipA | 16.38 | 67.36 |
| 15 | P0A817 | S-adenosylmethionine synthase | 15.29 | 41.95 |
| 16 | P23538 | Phosphoenolpyruvate synthase | 12.22 | 87.43 |
| 17 | P0AFG3 | 2-oxoglutarate dehydrogenase E1 component | 11.97 | 105.06 |
| 18 | P0A7G6 | Protein RecA | 11.96 | 37.97 |
| 19 | P60438 | Large ribosomal subunit protein uL3 | 11.69 | 22.24 |
| 20 | P04805 | Glutamate--tRNA ligase | 11.44 | 53.82 |
| 21 | P25526 | Succinate-semialdehyde dehydrogenase [NADP(+)] GabD | 11.01 | 51.72 |
| 22 | P0A7W7 | Small ribosomal subunit protein uS8 | 10.48 | 14.13 |
| 23 | P02359 | Small ribosomal subunit protein uS7 | 10.17 | 20.02 |
| 24 | P19318 | Respiratory nitrate reductase 2 beta chain | 9.71 | 58.56 |
| 25 | P0A6N4 | Elongation factor P | 8.41 | 20.59 |
| 26 | P0A7E5 | CTP synthase | 8.40 | 60.37 |
| 27 | P21170 | Biosynthetic arginine decarboxylase | 8.17 | 73.90 |
| 28 | P0A705 | Translation initiation factor IF-2 | 8.08 | 97.35 |
| 29 | P0A6H5 | ATP-dependent protease ATPase subunit HslU | 8.04 | 49.59 |
| 30 | P0A9Q5 | Acetyl-coenzyme A carboxylase carboxyl transferase subunit beta | 7.60 | 33.32 |
| 31 | P0A7D7 | Phosphoribosylaminoimidazole-succinocarboxamide synthase | 7.54 | 27.00 |
| 32 | P77316 | Uncharacterized zinc-type alcohol dehydrogenase-like protein YbdR | 7.36 | 44.18 |
| 33 | P0A6B7 | Cysteine desulfurase IscS | 7.19 | 45.09 |
| 34 | P0A715 | 2-dehydro-3-deoxyphosphooctonate aldolase | 7.19 | 30.83 |
| 35 | P08997 | Malate synthase A | 6.66 | 60.27 |
| Index | Accession | Protein Description | Score | Molecular Weight (kDa) |
| 36 | P0A7X3 | Small ribosomal subunit protein uS9 | 6.55 | 14.86 |
| 37 | P0A9W3 | Energy-dependent translational throttle protein EttA | 6.45 | 62.44 |
| 38 | P27550 | Acetyl-coenzyme A synthetase | 6.44 | 72.09 |
| 39 | P38097 | Probable diguanylate cyclase DgcE | 6.42 | 123.89 |
| 40 | P0A7R1 | Large ribosomal subunit protein bL9 | 6.38 | 15.77 |
| 41 | P0A8M0 | Asparagine--tRNA ligase | 6.33 | 52.57 |
| 42 | P46889 | DNA translocase FtsK | 6.33 | 146.66 |
| 43 | P21599 | Pyruvate kinase II | 6.29 | 51.36 |
| 44 | P0AFZ3 | Stringent starvation protein B | 6.28 | 18.26 |
| 45 | P0AG30 | Transcription termination factor Rho | 6.22 | 47.00 |
